# Comparative Transcriptome Analysis Reveals the Biocontrol Mechanism of *Bacillus velezensis* F21 Against *Fusarium* Wilt on Watermelon

**DOI:** 10.3389/fmicb.2019.00652

**Published:** 2019-04-03

**Authors:** Chun-Hao Jiang, Xie-Feng Yao, Dan-Dan Mi, Zi-Jie Li, Bing-Ye Yang, Ying Zheng, Yi-Jun Qi, Jian-Hua Guo

**Affiliations:** ^1^Department of Plant Pathology, College of Plant Protection, Nanjing Agricultural University – Key Laboratory of Monitoring and Management of Crop Diseases and Pest Insects, Ministry of Agriculture – Engineering Center of Bioresource Pesticides in Jiangsu Province, Nanjing, China; ^2^State Key Laboratory of Crop Genetics and Germplasm Enhancement, College of Life Sciences, Nanjing Agricultural University, Nanjing, China; ^3^Jiangsu Key Laboratory for Horticultural Crop Genetic Improvement, Institute of Vegetable Crops, Jiangsu Academy of Agricultural Sciences, Nanjing, China; ^4^Tsinghua Peking Center for Life Sciences, Center for Plant Biology, School of Life Sciences, Tsinghua University, Beijing, China

**Keywords:** watermelon, *Fusarium oxysporum* f. sp. *niveum* (Fon), biological control, *Bacillus velezensis*, induced systemic resistance, transcriptome profiling

## Abstract

The watermelon (*Citrullus lanatus*) is one of the most important horticultural crops for fruit production worldwide. However, the production of watermelon is seriously restricted by one kind of soilborne disease, *Fusarium* wilt, which is caused by *Fusarium oxysporum* f. sp. *niveum* (Fon). In this study, we identified an efficient PGPR strain *B. velezensis* F21, which could be used in watermelon production for Fon control. The results of biocontrol mechanisms showed that *B. velezensis* F21 could suppress the growth and spore germination of Fon *in vitro*. Moreover, *B. velezensis* F21 could also enhance plant basal immunity to Fon by increasing the expression of plant defense related genes and activities of some defense enzymes, such as CAT, POD, and SOD. To elucidate the detailed mechanisms regulating *B. velezensis* F21 biocontrol of *Fusarium* wilt in watermelon, a comparative transcriptome analysis using watermelon plant roots treated with *B. velezensis* F21 or sterile water alone and in combination with Fon inoculation was conducted. The transcriptome sequencing results revealed almost one thousand ripening-related differentially expressed genes (DEGs) in the process of *B. velezensis* F21 triggering ISR (induced systemic resistance) to Fon. In addition, the Gene Ontology (GO) classification and Kyoto Encyclopedia of Genes and Genomes (KEGG) pathway enrichment indicated that numerous of transcription factors (TFs) and plant disease resistance genes were activated and validated by using quantitative real-time PCR (qRT-PCR), which showed significant differences in expression levels in the roots of watermelon with different treatments. In addition, genes involved in the MAPK signaling pathway and phytohormone signaling pathway were analyzed, and the results indicated that *B. velezensis* F21 could enhance plant disease resistance to Fon through the above related genes and phytohormone signal factors. Taken together, this study substantially expands transcriptome data resources and suggests a molecular framework for *B. velezensis* F21 inducing systemic resistance to Fon in watermelon. In addition, it also provides an effective strategy for the control of *Fusarium* wilt in watermelon.

## Introduction

The watermelon (*Citrullus lanatus*) is one of the most important horticultural crops for fruit production in the world and is known as the most popular fruit in the summer. In fact, according to research statistics, watermelon accounts for almost 2% of the world’s total area devoted to vegetable production. The cultivated area and yield of watermelon have increased yearly due to increasing consumption and its comparatively high economic value in China (Sun et al., 2017). However, the production of watermelon is seriously restricted by soilborne diseases, such as *Fusarium* wilt, anthracnose, and gummy stem blight and so on. Watermelon *Fusarium* wilt, caused by Fon, is a devastating disease affecting a large variety of economically important crops worldwide (Demers et al., 2015). The symptoms of *Fusarium* wilt disease are necrotic lesions, followed by foliar wilting as the pathogen invades the vascular system of plants, and eventually plant death symptoms can manifest in a few weeks (Janvier et al., 2007). The disease can result in 100% yield losses under favorable environmental conditions during the watermelon production. Due to the long-term survival and accumulation of pathogens in soil, there is currently no effective control strategy. In addition, as a result of the substantial loss incurred by the devastating pathogen, effective non-chemical, environmentally friendly strategies to prevent or reduce the damage caused by Fon in watermelon production are valuable and urgently needed.

Biological control of soilborne diseases has been studied for over 100 years, and it has been considered a viable alternative method to chemical control (Heydari and Pessarakli, 2010). It is one of the highest potential strategies for soilborne disease control due to its advantages of environmental friendliness, safety and lack of the induction of pesticide resistance occurring (Jiang et al., 2015). To date, studies on the biological control of watermelon *Fusarium* wilt are still in progress and an efficient strategy for Fon control is still in discovery. For instance, *Paenibacillus polymyxa* SQR-21 has been identified by Ling et al. (2011) as a potential agent for the biocontrol of *Fusarium* wilt in watermelon. They demonstrated that *P. polymyxa* SQR-21 could systemically affect root exudates of watermelon to decrease the conidial germination of Fon. In addition, the substantial potential of species of *Streptomyces* to suppress plant diseases caused by fungal pathogens has also been reported in recent years. For example, the strain *S. goshikiensis* YCXU was evaluated as an efficient biocontrol agent (BCA) that showed antifungal activity against Fon by producing CAT, chitinase, β-1,3-glucanase and urease enzymes (Faheem et al., 2015). Lu et al. (2016) reported that a new species of *Streptomyces*, *S. rimosus* M527, was isolated and identified as a BCA against *F. oxysporum* f. sp. *cucumerinum*. They demonstrated that M527 not only displayed broad-spectrum antifungal activity but also showed the strongest antagonistic activity against the spore germination of *F. oxysporum* f. sp. *cucumerinum* (Lu et al., 2016). Moreover, *Bacillus* spp. have been investigated for their potential ability to serve as a BCA against a variety of plant diseases (Zhao et al., 2014). The high potential of *B. amyloliquefaciens* SN16-1 to control *F. oxysporum* f. sp. *lycopersici* was recently reported (Wan et al., 2017). Yang et al. (2014) found that *B. subtilis* B006 could manage cucumber wilt disease through the suppression of *Fusarium oxysporum* f. sp. *cucumerinum* in rhizosphere. In addition to the biocontrol bacteria listed above, many other species are also being discovered that serve as BCAs for *Fusarium* wilt disease control in watermelon.

In recent years, the research on the biological control of *F. oxysporum* is has been devoted not only to the discovery of new BCAs but also to the biocontrol mechanisms of BCAs. So far this year, a few reports on the biocontrol mechanisms of BCAs against *Fusarium* wilt have been reported. The biocontrol mechanisms operating against *Fusarium* wilt include the production of diffusible and volatile antimicrobial compounds, changes in soil microbial diversity and ISR. Wang et al. (2013) reported that the antagonistic effect of *B. amyloliquefaciens* W19 is the main mechanism of banana *Fusarium* wilt biocontrol. Three kinds of antifungal lipopeptides (iturin, surfactin, and bacillomycin D) secreted by W19 were identified in their study. In addition, more than 18 volatile antifungal compounds with significant antagonistic effects against *F. oxysporum* were identified (Wang et al., 2013). Otherwise, the microbiome of plants playing a crucial role in both plant and ecosystem health, and the maintenance of microbial diversity in the soil can promote plant growth, prevent diseases, and increase stress resistance. Berg et al. (2017) reported that microbial diversity was a key factor in preventing diseases and could be implemented as a biomarker in plant protection strategies. Shen et al. (2014) reported that the application of BIO could suppress the bacterial community associated with banana *Fusarium* wilt disease. They found that the abundances of Gemmatimonadetes and Acidobacteria were high in BIO-treated soil, while that of Bacteroidetes was low (Shen et al., 2014). In addition, it is envisaged that in suppressive soils plant roots are associated with microbial communities that have an overall beneficial effect on plant health. This phenomenon is known as ISR triggered by plant beneficial microbes (Choudhary et al., 2007). Many effective biocontrol PGPR can elicit ISR and related research results have been reported in recent years. For example, the *Pseudomonas fluorescens* WCS417r-mediated ISR has been shown to function against various plant pathogens in Arabidopsis by activating the JA signaling pathway, there by affecting such pathogens as the bacterial leaf pathogen *P. syringae* pv. *tomato* and the fungal root pathogen *Fusarium oxysporum* f. sp. *raphani* (Pieterse et al., 1998). Niu et al. (2011) first demonstrated that the PGPR *Bacillus cereus* AR156 can induce systemic resistance in *Arabidopsis thaliana* by simultaneously activating SA-and JA/ETH-dependent signaling pathways. In subsequent studies, two TFs, WRKY11 and WRKY70, were identified as important regulators involved in the ISR triggered by *B. cereus* AR156 (Jiang et al., 2016). Current research shows that ISR is also one of the important mechanisms of *Fusarium* biological control. It has been reported that plant growth-promoting rhizobacteria (PGPR) *B. fortis* and *B. subtilis* have ISR against *Fusarium* wilt disease in controlled environments (Akram et al., 2013). Bekkar et al. (2018) found that *Bacillus* strains application could induce systemic resistance against *Fusarium* wilt in chickpeas. Therefore, in-depth analysis of ISR triggered by PGPR is of great significance to the biological control of *Fusarium* wilt.

In this study, we identified an efficient PGPR strain *B. velezensis* F21 which can be used for *F. oxysporum* f. sp. *niveum* control in watermelon. *B. velezensis* F21 can not only promote watermelon growth but also protect watermelon from Fon invasion. These results indicated that *B. velezensis* F21 could suppress the growth and spore germination of Fon *in vitro*. Moreover, the PGPR strain could enhance plant basal immunity to Fon by increasing the expression of plant defense related genes and activities of some defense enzymes. Otherwise, the transcriptome sequencing is a highly efficient, widely used, molecular biological research method used to obtain detailed transcriptome information. In this study, a total of 20,985 genes in watermelon were identified and the transcriptome analysis revealed almost 1,000 ripening-related DEGs in the process of *B. velezensis* F21 triggering ISR to Fon. These genes were subjected to scatter plot analysis, the GO classification and KEGG pathway enrichment. Furthermore, a series of TFs and plant disease resistance genes (PRGs) were identified and validated by using quantitative real-time PCR (qRT-PCR), which showed significantly different expression levels in the roots of watermelon with different treatments. Finally, we analyzed the genes involved in the MAPK signaling pathway and plant hormone signaling pathways and validated the gene expression level and hormone accumulation content by qRT-PCR and ELISA, respectively. Collectively, this study suggests a molecular framework for the induction of systemic resistance to Fon in watermelon by *B. velezensis* F21, and provides detailed transcriptome data resources for further molecular mechanistic research.

## Materials and Methods

### Plant Material, Bacteria, Fungi, Growth Condition, and Experimental Design

In this study, we used one kind of watermelon cultivar (Sumi #1) as a test plant. The variety was susceptible to *F. oxysporum* f. sp. *niveum.* It originated from the Institute of Vegetable Crops, Jiangsu Academy of Agricultural Sciences, China.

All the PGPR that we used in this study came from our biocontrol bacteria library, which consisted of beneficial bacteria isolated by our lab from the field where *Fusarium* wilt previously occurred in different provinces of China. All the bacteria were grown on LB agar media and incubated at 28°C for 24 h. One single colony on the medium was picked and then inoculated into LB broth and incubated at 28°C for 24 h while shaking at 200 rpm. The bacterial fermented liquid was centrifuged at 6,000 rpm for 10 min, and the supernatant was discarded, suspended in sterile water and adjusted to a concentration of 1 × 10^8^ CFU/mL for further experiments.

*F. oxysporum* f. sp. *niveum*, *F. oxysporum* f. sp. *cubense*, *F. graminearum, Mycosphaerella melonis*, and *B. cinerea* were used in antagonistic *in vitro* studies. They were cultured on potato dextrosa agar (PDA) medium at 25°C under dark conditions for 5 days. The microconidial suspension of *F. oxysporum* f. sp. *niveum* used in spore germination and greenhouse experiments was prepared as described by Meldrum et al. (2013).

### Effects of *B. velezensis* F21 on Hydrolase Activity, Fungal Antagonism, and Spore Germination

To evaluate the biocontrol potential of *B. velezensis* F21, we evaluated the effect of some hydrolase activities and fungal antagonism. For the hydrolase activity evaluation, we detected the activities of protease, chitinase, ferric enzyme, glucanase, and cellulose as described by Jiang et al. (2015). For the fungal antagonism, we adopted a flat confrontation experiment on PDA medium *in vitro*. The Five-millimeter plugs from 5-day-old cultures of Fon were inoculated in the center of the PDA medium, and then *B. velezensis* F21 was inoculated on both sides of the culture dish by using filter paper. Sterile water was used as a control. Subsequently, all the plates were incubated at 25°C (Jiang et al., 2018), and the colony diameters were measured and recorded every 12 h after. To verify the broad-spectrum effect of *B. velezensis* F21, we also used other pathogenic fungi besides Fon, such as *F. oxysporum* f. sp. *cubense*, *F. graminearum, Mycosphaerella melonis*, and *Botrytis cinerea*.

Spore germination assays were performed as described by Mujoko et al. (2014) with minor modifications. The *B. velezensis* F21 culture in agar media was fragmented by a cork borer, and the top layer was sliced. Then, the top surface of the slice containing *B. velezensis* F21 was removed and assumed to contain metabolites produced by *B. velezensis* F21. The slice was transferred into deck glass for spore germination observation. The agar disk was inoculated with 10 μl Fon conidia suspension at a concentration of 1 × 10^5^ conidia/ml, kept in the slide box and incubated for 5 h at 25°C. The germination of conidia was observed and photographed using an Olympus DP71 camera with an Olympus BX51-P microscope (Olympus America, Inc.).

### Experimental Design in Greenhouse and Filed Trials

To evaluate the plant growth-promoting effect of *B. velezensis* and the biocontrol effect on watermelon *Fusarium* wilt, greenhouse and field experiments were carried out in this study. For the greenhouse experiment, the watermelon cultivar (Sumi #1) was used as the test plant. The seeds of watermelon were sown into sterile pots with one seedling per pot and then cultivated in a greenhouse with a 14 h day (200 μE m^-2^ s^-1^ at 28°C) and a 10 h night (at 20°C) cycle at 70% relative humidity. The seedlings were treated with *B. velezensis* F21 by sprinkling the root in combination with spraying the leaf when the seedlings grew to three leaves, and sterile water served as a control. Five days later, the watermelon seedlings in each treatment were challenged with Fon by inoculation with spore suspensions in 5 days, and the inoculation rate was 20 ml (1 × 10^5^ conidia/ml) per seedling. The *Fusarium* wilt symptoms were recorded and photographed 15 days after inoculation. In addition, the plant materials at different time points were harvested for RNA sequencing, plant defense related gene expression and related enzyme activity analysis. The plant growth-promoting effect of *B. velezensis* F21 on watermelon was also evaluated in this study, and the results were recorded and photographed after 30 days of *B. velezensis* F21 treatment.

Moreover, the plant growth promotion effect of *B. velezensis* and the biocontrol effect on watermelon *Fusarium* wilt were also verified in the field. The field experiment was conducted in Guangzhou, China in watermelon growing seasons by using one kind of watermelon cultivar (Sumi #1). The trial experiment was carried out on private land: the field was located in Guangzhou Province, China, with GPS coordinates of N 23°23′28.68″, E113°26′7.14″ and the field studies did not involve endangered or protected species. Two treatments were established, and water was used as a mock control. Twenty milliliters of a 1 × 10^8^ CFU/mL suspension of *B. velezensis* F21was poured onto the roots and both sides of the leaves were sprayed with the suspension when the watermelon seedlings grew to one real leaf status. Sixty days later, the growth-promoting effect on watermelon and disease occurrence were recorded.

### RNA Extraction, Gene Expression, and Activities of Plant Defense-Related Enzyme Analysis

The roots of watermelon in each treatment for qRT-PCR analysis were harvested and soaked in liquid nitrogen. The total RNA of plants was extracted by using the TRIZOL reagent (Invitrogen, Cat^#^: 15596-026), following the manufacturer’s recommendations.

In this study, we analyzed the expression of DEGs and some genes related to the plant defense signaling pathway, such as the SA and JA signaling pathways. RT-PCR was developed with 0.5 mg total RNA, followed by DNase I treatment (gDNA Wiper from Vazyme^TM^, Cat^#^: R133-01). Then reverse transcription was conducted using HiScript^TM^ Q Select RT SuperMix (Vazyme^TM^, Cat^#^: R133-01). qRT-PCR was conducted on an ABI 7500 system (ABI, United States) by using the SYBR premix Ex-Taq mixture (Takara). The reaction was performed under the following conditions: 94°C for 5 min, followed by 45 cycles of 94°C for 10 s, 55°C for 20 s, and 72°C for 30 s, and end at 72°C for 5 min. The *actin* gene of watermelon (Cla008455) was employed as the internal standard. All the gene information and PCR primers used in our study are shown in Supplementary Table S1.

In addition, we also analyzed the activities of plant defense related enzymes (such as SOD, CAT, and POD) during the process of *B. velezensis* F21 triggering ISR to *Fusarium* wilt in watermelon. The activities of POD, SOD, and CAT in plant roots were measured by using the corresponding test kits as described by Jiang et al. (2015), who used a ‘POD detection kit,’ ‘SOD detection kit,’ and ‘CAT detection kit,’ respectively, (Nanjing Jiancheng Biological Engineering Institute, Nanjing, China) according to the manufacturer’s instructions.

### Library Construction, RNA Sequencing, and *de novo* Assembly

In the present study, a total of 20 samples were used for library construction. Twelve of the samples were obtained from watermelon treated with *B. velezensis* F21 or sterile water alone at 0 dpt, 3 dpt, 5 dpt, 8 dpt, 11 dpt, and 13 dpt. Moreover, the remaining eight samples obtained from watermelon pretreated with BCA and then combined with Fon challenge at 0 dpt, 3 dpt, 5 dpt, and 8 dpt. All samples were harvested from at least five individual watermelon roots, and the mixture was quickly placed in liquid nitrogen. The total RNA of each sample was isolated according to the method described above.

The RNA sequencing library preparation was performed with 10 μg RNA sample by using a TruSeq RNA Sample Prep Kit (Illumina, San Diego, CA, United States) following the manufacturer’s instructions. Briefly, the mRNAs collected from the roots of watermelon with different treatments were purified, broken into short fragments and further used to synthesize first-strand cDNAs with hexamer and reverse transcriptase (Promega). Subsequently, the double-stranded cDNA was synthesized with DNA polymerase I and RNase H. Then the final cDNA library was generated using Phusion High-Fidelity DNA polymerase (Thermo Scientific^TM^, United States, CA#: F530L) after the end of the repairment process with a single adenine residue and adapter ligation. The quality of the library was evaluated by using bioanalyzer plots. Illumina sequencing was carried out in the Beijing Genomics Institute (BGI), China, and performed using the HiSeq^TM^ 2500 platform according to the manufacturer’s instructions (Illumina, San Diego, CA, United States). The sequencing data were saved as FASTQ files, and deposited to National Center for Biotechnology Information (NCBI) (BioProject accessions: PRJNA503589; BioSample accessions: SAMN10365249, SAMN10365250, SAMN10365251, SAMN10365252, SAMN10365253, SAMN10365254, SAMN10365255, SAMN10365256, SAMN10365257, SAMN10365258, SAMN10365259, SAMN10365260, SAMN10365261, SAMN10365262, SAMN10365263, SAMN10365264, SAMN10365265, and SAMN10365266).

### Raw Read Processing, *de novo* Assembly, and Functional Annotation

To obtain the sequencing information, the raw reads were initially processed by Illumina Pipeline Software. Then, the reads were cleaned by removing adaptor sequences and low-quality reads (*Q* < 20) with more than 10% uncertain (N) bases using in-house Perl scripts (Zhang et al., 2015). All the following analyses were based on the high-quality clean data we obtained. *De novo* assembly of the transcriptome was performed by using the trinity platform^1^ (Grabherr et al., 2011). After that, the data were mapped back onto the corresponding contigs with all the clean reads. Then, all contigs were assembled to produce unigenes with no extension on either end (Chen Y. et al., 2017). The assembled transcripts with significant gene expression values were subjected to a similarity search against the NCBI non-redundant protein database using the BlastX (E-value ≤ 10^-5^) program (Altschul et al., 1997). Blast annotations (NCBI id) were mapped back to the UniProt protein database and GO terms (molecular function, biological process, and cellular component) were extracted from the UniProt database. To identify more putative functions, all assembled unigenes were searched against several publicly available databases such as the NCBI non-redundant (NR) protein database, the Swiss-Prot protein database, the KEGG pathway database, and the GO database (Wu et al., 2016).

### Identification of Differentially Expressed Genes (DEGs), GO Enrichment, Pathway Analysis and Transcription Factor and PRG Identification

To quantify the identified gene expression, the number of fragments per kilobase of exon per million fragments (FPKM) mapped was used. The differential expression analysis of each sample was performed using the DESeq R package(1.10.1) and the DEGs were determined with a log-fold expression change (log FC) greater than 2 or less than -2 using a threshold of false discovery rates (FDR < 0.001) and a high statistically significant value (*P* < 0.05). GO and KEGG pathway enrichment analyses for all the DEGs were then carried out. The obtained GO annotation was enriched and refined using the GOseq R package with the “ELIM” method and Kolmogorov–Smirnov test. KEGG pathways were performed in KOBAS 2.0 plates and enriched by using in-house scripts according to Fisher’s exact test PRI. Enriched *p*-values were calculated and adjusted by using the Bonferroni correction. Then the corrected *p*-value of 0.05 was selected as the threshold to determine significant enrichment of the gene sets. Furthermore, to identify the TFs represented in all watermelon roots, all assembled unigenes were searched against the plant TF database (PlnTFDB^2^) by using a BLASTX search with the stringency of *E*-value 1e-06 (Moriya et al., 2007). Finally, to identify the plant resistance genes (PRGs) that function on *B. velezensis* F21 to trigger ISR to *Fusarium* wilt in watermelon, all the assembled unigenes were searched by using the PRG database (PRG-Wiki^3^).

### Validation of DEGs by qRT-PCR

To confirm the transcriptome data, 38 DEGs were randomly selected and verified (Supplementary Table S1). The expression of validated genes was analyzed by qRT-PCR on ABI 7500 Real-Time PCR System (Applied Biosystems, United States). The qRT-PCR procedure was performed as described previously. All the information of selected genes and primers designed by Primer premier 6.0 are shown in Supplementary Table S1. Finally, to ensure the reliability and reproducibility of the validation results, four technical replicates of each biological replicate and three independent biological replicates for each sample were arranged.

### Validation of Hormone Accumulation by Enzyme-Linked Immunosorbent Assay (ELISA)

In this study, the transcriptome results indicated that some plant hormone signaling pathways involved in *B. velezensis* F21 trigger ISR to *Fusarium* wilt in watermelon. In addition to validate the expression of genes involved in phytohormone synthesis and regulation, the accumulation of endogenous phytohormones, such as ABA, ETH, BR, JA, and SA, was also detected by ELISA with corresponding phytohormone ELISA test kits purchased from Nanjing Jin Yi Bai Biotechnology Co., Ltd.^4^ In this part, a total of 20 samples were used, and 12 of the samples were obtained from watermelon treated with *B. velezensis* F21 or sterile water alone at 0 dpt, 3 dpt, 5 dpt, 8 dpt, 11 dpt, and 13 dpt. Moreover, the remaining eight samples obtained from watermelon pretreated with a BCA and then combined with a Fon challenge at 0 dpt, 3 dpt, 5 dpt, and 8 dpt. All samples were harvested from at least three individual watermelon roots, and the mixture was quickly placed in liquid nitrogen. The detailed method for phytohormone extraction and quantification refer to the respective ELISA kit instructions. Moreover, for each treatment, four technical replicates of each biological replicate and three independent biological replicates for each sample were arranged to ensure the reliability and reproducibility of validation results.

### Data Analysis

All bioassays and experiments were conducted three times with 36 seedlings per treatment. Significant differences between means were compared by using the LSD test (Fisher’s protected least-significant differences test) at *P* = 0.05. A *P*-value of <0.05 was considered statistically significant. All statistical analyses were performed by using analysis of variance in SPSS 24.0 (IBM SPSS Inc., United States).

## Results

### *B. velezensis* F21 Could Serve as a Potential Biocontrol Agent to Control *Fusarium* Wilt Disease in Watermelon

**FIGURE 1:**
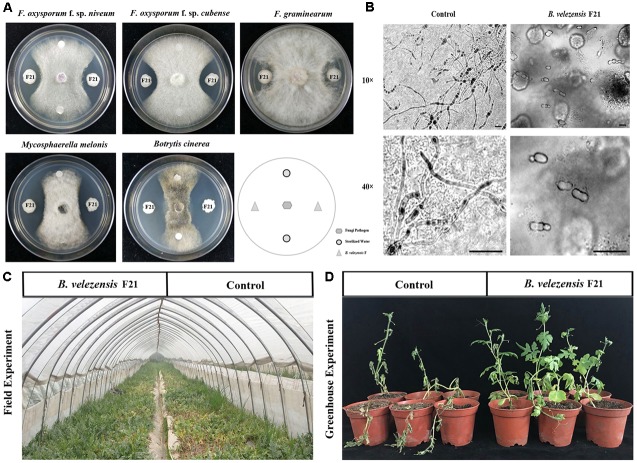
*B. velezensis* F21 served as a potential biocontrol agent for control of *Fusarium* wilt disease in watermelon. *B. velezensis* F21 can significantly control watermelon *Fusarium* wilt disease. **(A)** Antagonistic effect of biocontrol bacteria on various pathogenic fungi, such as *Fusarium oxysporum* f. sp. *niveum*, *Fusarium oxysporum* f. sp. *cubense*, *Fusarium graminearum*, *Mycosphaerella melonis*, and *Botrytis cinerea*. **(B)** Effect of *B. velezensis* F21 on spore germination were observed by microscope, the bars equal 5 μm. **(C)** Biological control effect of *B. velezensis* F21 to *Fusarium* wilt disease on watermelon in the field. **(D)** The symptoms of *Fusarium* wilt disease development on watermelon seedlings 30 days post *Fusarium oxysporum* f. sp. *niveum* inoculation in each treatment in greenhouse experiment.

*B. velezensis* F21 was isolated by using the antagonistic and hydrolase activity examination assessment system previously established by our laboratory (Jiang et al., 2018). The results indicated that *B. velezensis* F21 had antagonistic activity against Fon *in vitro* (Figure 1A). Moreover, we found that the antagonism of *B. velezensis* F21 to fungal pathogens might be broad-spectrum. The strain also showed antagonistic effects against other fungal pathogens, such as *F. oxysporum* f. sp. *cubense*, *F. graminearum*, *M. melonis*, and *B. cinerea* (Figure 1A). In addition, it was also found that *B. velezensis* F21could inhibit the germination of *F. oxysporum* spores (Figure 1B). The results of hydrolase activity examination indicated that *B. velezensis* F21 had multiple hydrolase activities, such as protease, ferric enzyme, glucanase and cellulose, but no chitinase activity (Supplementary Figure S1). Based on the above results, we found that *B. velezensis* F21 had the potential to control watermelon *Fusarium* wilt. To verify this hypothesis, we conducted greenhouse experiments and field experiments and the results indicated that *B. velezensis* F21 could significantly control *Fusarium* wilt disease in the greenhouse and in the field. The symptoms of watermelon *Fusarium* wilt in the *B. velezensis* F21 treatment were significantly weaker in compared with the control treatment (Figure 1C,D) and the biocontrol efficiency of *B. velezensis* F21 in the greenhouse and in the field was 80.35% and 65.81%, respectively (Supplementary Table S2).

As is well known, an efficient BCA also plays a role in promoting plant growth. Therefore, in this study, we also evaluated the plant growth promoting effect of *B. velezensis* F21 on watermelon through greenhouse and field experiments. As shown in Supplementary Figure S2, the growth state of watermelon treated with *B. velezensis* F21 was significantly better than that of the control treatment, and the field experiment results still showed that the size and yield of watermelon were significantly higher than those of the control treatment (Supplementary Figure S2). Taken together, it could be concluded that *B. velezensis* F21 could serve as a potential BCA to control *Fusarium* wilt disease in watermelon.

### *B. velezensis* F21 Treatment Triggers ISR to Fon by Inducing Defense-Related Gene Expression and Raising the Activities of Plant Defense-Related Enzymes in Watermelon

**FIGURE 2:**
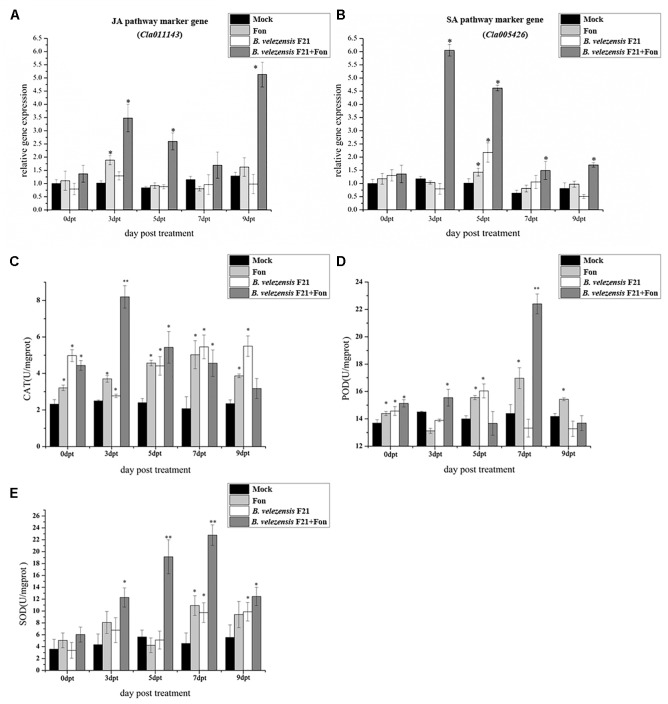
Effect on the expression of defense related gene and activities of defense-related enzymes in the process of controlling watermelon *Fusarium* wilt by *B. velezensis* F21. **(A,B)** Expression of defense related gene in watermelon root in response to interaction between *B. velezensis* F21 and *F. oxysporum* f. sp. *niveum.* Time course of expression of JA signaling pathway marker gene *Cla011143* **(A)** and SA signaling pathway marker gene *Cla005426* **(B)** in the root of watermelon treated with *B. velezensis* F21and challenged with Fon. The expression values of the individual genes were normalized using *Actin* gene as an internal standard. **(C–E)** The activities of defense-related enzymes (CAT, POD, and SOD) in the root of watermelon pretreated with *B. velezensis* F21 and challenged with Fon. All data were presented as means of three replicates ± SD, and error bars represent SD for three replicates. Means with asterisk have significant differences (^∗^*P* < 0.05 and ^∗∗^*P* < 0.01; LSD test). All experiments were performed three times, and similar results were obtained.

Biocontrol agent-mediated ISR is often combined with priming for the enhanced expression of defense-related genes and the activities of defense-related enzymes. To determine the mechanism of *B. velezensis* F21-mediated ISR in *Fusarium* wilt, the expression levels of defense related genes, such as *Cla011143* (JA pathway marker gene) and*Cla005426* (SA pathway marker gene) were detected. Moreover, the activities of defense related enzymes (CAT, POD, and SOD) were also determined in the roots of watermelon only inoculated with Fon and in those pretreated with *B. velezensis* F21 before inoculation with Fon. As shown in Figure 2A,B, the transcripts of *Cla01114* and *Cla005426* accumulated in the roots of watermelon from 0 dpt to 9 dpt. The transcripts of two tested genes were both generally stronger in the roots of watermelon pretreated with *B. velezensis* F21 and then challenged with Fon from 3 dpt to 9 dpt and reached their maximum at 3 dpt in comparison with the control treatment. In addition, we also found that *B. velezensis* F21 could result in a quicker response of related genes (Figure 2A,B). These results indicated that *B. velezensis* F21 could prime the watermelon to enhance resistance to Fon by simultaneously activating the SA-and JA-mediated defense signaling pathways. Moreover, the activities of defense related enzymes (CAT, POD, and SOD) were also determined and the results in Figure 2 demonstrated that compared with the control treatment, *B. velezensis* F21 could increase the activities and advance those defense-related enzymes to maximum activity in advance in response to Fon invasion (Figure 2C–E). As shown in Figure 2, the activities of CAT, POD, and SOD in the roots of watermelon pretreated with *B. velezensis* F21 combined with the challenge with Fon were higher and showed a high peak at 3 dpt, 7 dpt, and 7 dpt, respectively (Figure 2C–E).

### Identification and Functional Annotation of Differentially Expressed Genes (DEGs) During the Process of *B. velezensis* F21 Biocontrol of *Fusarium* Wilt in Watermelon

To attempt to elucidate the detailed mechanisms of *B. velezensis* F21 in the biocontrol of *Fusarium* wilt in watermelon, a comparative transcriptome analysis using watermelon plant roots treated with *B. velezensis* F21 or sterile water alone and in combination with Fon inoculation was conducted. All libraries from different samples were sequenced by using a HiSeq^TM^ 2500 platform. Approximately 21.61 Gb total clean reads were obtained after cleaning and quality checking. The Q20 percentage of each library was from 97 to 99.11%; the Q30 percentage of each library was from 89 to 95%; and the clean reads ratio percentage of each library was up to 99% (Supplementary Table S3). These data showed that the RNA-Seq quality was applicable for further analysis.

**FIGURE 3:**
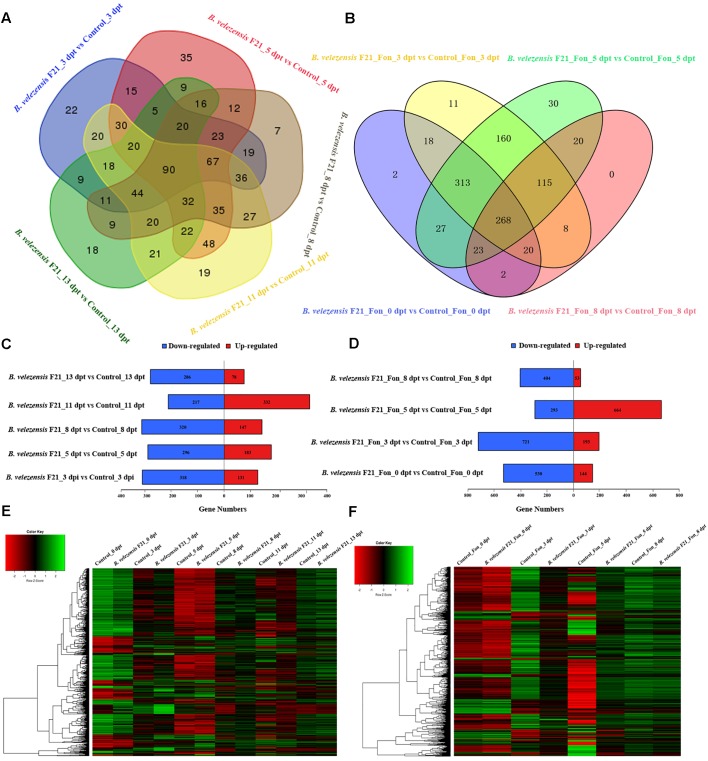
Classification of differential expressed genes (DEGs) in the root of watermelon in response to *B. velezensis* F21 treatment or in combination with Fon inoculation. **(A)** Venn diagram of DEGs in the root of watermelon treated by *B. velezensis* F21 alone at different time points. **(B)** Venn diagram of DEGs in the root of watermelon pretreated with *B. velezensis* F21 and challenged with Fon at different time points. **(C,D)** Numbers of DEGs in response to *B. velezensis* F21 treatment or in combination with Fon inoculation. *X*-axis represents DEG numbers, *Y*-axis represents comparison method between each group. Red color represents up-regulated DEGs, blue color represents down-regulated DEGs. **(E)** Cluster analysis of DEGs in root of watermelon treated with *B. velezensis* F21 alone and in root of mock-treated watermelon seedlings based on the expression profiles measured by RNA-seq. **(F)** Cluster analysis of DEGs in root of watermelon pretreated with *B. velezensis* F21 and challenged with Fon based on the expression profiles measured by RNA-seq. The color scale in the heat map corresponds to log_2_ (FPKM) value of genes in each samples.

To delineate the mechanisms of *B. velezensis* F21 biocontrol *Fusarium* wilt in watermelon, two comparison groups were set with sequencing data from the 20 libraries. One group was the “pretreatment” group, using *B. velezensis* F21-treated samples alone compared with mock treatment samples at different time points and six sets of comparisons were made. The other group was the “ISR” group by using *B. velezensis* F21 pretreated samples for 5 days and then challenging the plants with Fon compared with control treatment samples at different time points and four sets of comparisons were made. It was observed that whether *B. velezensis* F21 was treated alone or pretreated before inoculating the pathogen, a large number of gene expression levels in watermelon could be changed (Supplementary Table S4 and Figure 3A,B). The comparative results revealed that 768 significant DEGs were identified in the samples treated with *B. velezensis* F21 alone and the gene transcription levels were most affected at 11 dpt, with 549 DEGs (217 down-regulated and 332 up-regulated) (Figure 3C,E). However, during the process of *B. velezensis* F21 ISR to Fon, over 1,000 significant DEGs were identified and the gene transcription levels were influenced most strongly at 3 dpt and 5 dpt. There were 894 DEGs (721 down-regulated and 193 up-regulated) and 957 DEGs (293 down-regulated and 664 up-regulated), respectively (Figure 3D,F). The results indicated that *B. velezensis* F21 could result in a quicker and stronger watermelon response to Fon invasion. In summary, the results showed that *B. velezensis* F21 could succeed in inducing the resistance of watermelon to *Fusarium* wilt by regulating the transcription of some related genes.

**FIGURE 4:**
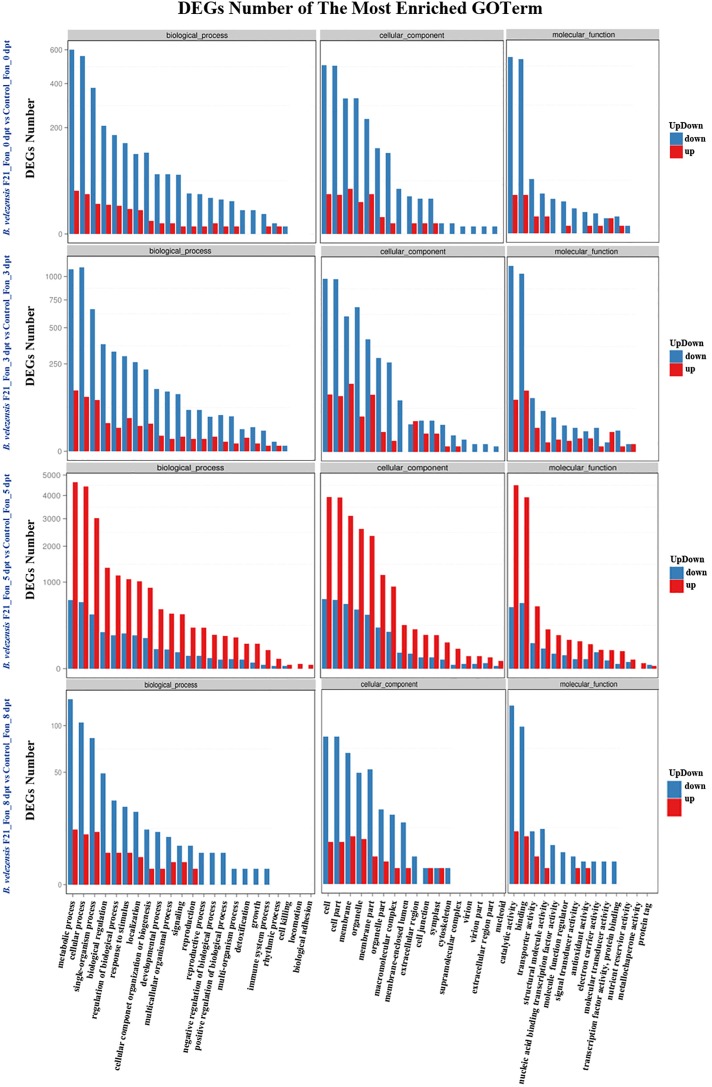
GO classification of up-regulated and down-regulated genes. GO classification of DEGs in the comparisons of *B. velezensis* F21 pretreated or mock treated watermelon seedlings, both challenged with Fon at different time points. *X*-axis represents GO term. *Y*-axis represents the amount of up- and down-regulated genes.

Otherwise, the global functional analysis of DEGs was carried out by using GO annotation to derive “biological process,” “cellular component,” and “molecular function” (Supplementary Figure S3 and Figure 4). As shown in Supplementary Figure S3 and Figure 4, in comparison with mock treatment or treatment inoculated with Fon alone, the most enriched terms for “biological process” were “metabolic process,” “cellular process,” and “single-organism process” in the libraries constructed from watermelon plants treated with *B. velezensis* F21 alone or pretreated with *B. velezensis* F21 and then inoculated with Fon. Moreover, the “cell,” “cell part,” and “membrane” were most enriched in the term “cellular component.” “The catalytic activity” term was the most frequent in the “molecular function,” followed by “transporter activity” and “binding” (Supplementary Figure S3 and Figure 4). The above results show that *B. velezensis* F21 could change the physiological and biochemical functions of watermelon during biological control in *Fusarium* wilt.

### Pathway Enrichment Analysis for DEGs Identified in the Process of *B. velezensis* F21 Biocontrol *Fusarium* Wilt Disease

**FIGURE 5:**
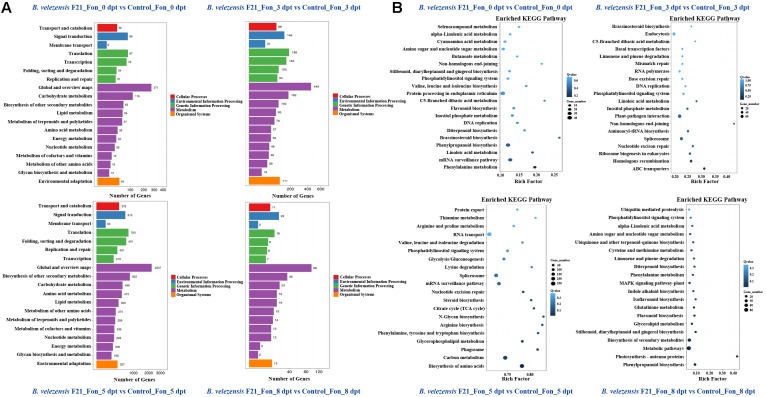
Kyoto Encyclopedia of Genes and Genomes (KEGG) pathway classification and functional enrichment of DEGs. **(A)** Pathway classification of DEGs in the comparisons of *B. velezensis* F21 pretreated or mock treated watermelon seedlings then both challenged with Fon at different time points, *X*-axis represents number of DEG. *Y*-axis represents functional classification of KEGG. There are seven branches for KEGG pathways: cellular processes, environmental information processing, genetic information processing, human disease (for animals only), metabolism, organismal systems and drug development. **(B)** Pathway functional enrichment of DEGs in the comparisons of *B. velezensis* F21 pretreated or mock treated watermelon seedlings, both challenged with Fon at different time points, *X*-axis represents enrichment factor. *Y*-axis represents pathway name. The color indicates the *q*-value (high: white, low: blue), the lower *q*-value indicates the more significant enrichment. Point size indicates DEG number (the bigger dots refer to larger amount). Rich Factor refers to the value of enrichment factor, which is the quotient of foreground value (the number of DEGs) and background value (total gene amount). The larger the value, the more significant enrichment.

Pathway enrichment analysis with DEGs could provide guidance to identify significant metabolic pathways. To further investigate the biochemical pathways of these DEGs in the process of *B. velezensis* F21 ISR to Fon, we mapped all DEGs identified in the RNA sequencing to terms in the KEGG database. As shown in Supplementary Figure S4 and Figure 5A, the DEGs affected by *B. velezensis* F21 could be classified into five pathways, including cellular processes, environmental information processing, genetic information processing, metabolic and organism systems, whether the watermelon was treated by *B. velezensis* F21 alone or pretreated with *B. velezensis* and then challenged with Fon. Moreover, the metabolic pathway contained the largest number of DEGs, followed by genetic information processing (Supplementary Figure S4 and Figure 5A). In addition, we found that a 5-day treatment of *B. velezensis* F21 alone for 5 days had the most significant effect on the metabolic pathways in watermelon Supplementary Figure S4. While in the process of biological control of *Fusarium* wilt at 5 days post pathogen inoculation, the effects on plant metabolic pathways and the number of DEGs reached their maximum (Figure 5A). In addition, we also analyzed the enrichment in the KEGG pathway with DEGs to predict the biochemical pathway. The top 20 enriched KEGG pathways associated with DEGs affected by *B. velezensis* F21 at different timepoints are shown in Supplementary Figure S5. It was demonstrated that some KEGG pathways associated with plant immunity were most enriched, such as the MAPK signaling pathway, plant–pathogen interaction, and plant hormone signaling transduction (Supplementary Figure S5). The results indicated that *B. velezensis* F21 treatment could enhance plant basal immunity. Otherwise, we also analyzed the enrichment in the KEGG pathway with DEGs identified in the process of *B. velezensis* F21 ISR to Fon. Likewise, the top 20 enriched KEGG pathways associated with DEGs influenced by *B. velezensis* F21 at different timepoints are shown in Figure 5B. From the results, we found that KEGG pathways, such as plant–pathogen interaction, ABC transporter plant hormone signaling transduction, and MAPK signaling pathways play important roles in the process of *B. velezensis* F21 ISR to Fon (Figure 5B).

### Transcription Factors and Plant Disease Resistance Genes Prediction of DEGs Consistent With *B. velezensis* F21-Triggered Immunity to Fon

**FIGURE 6:**
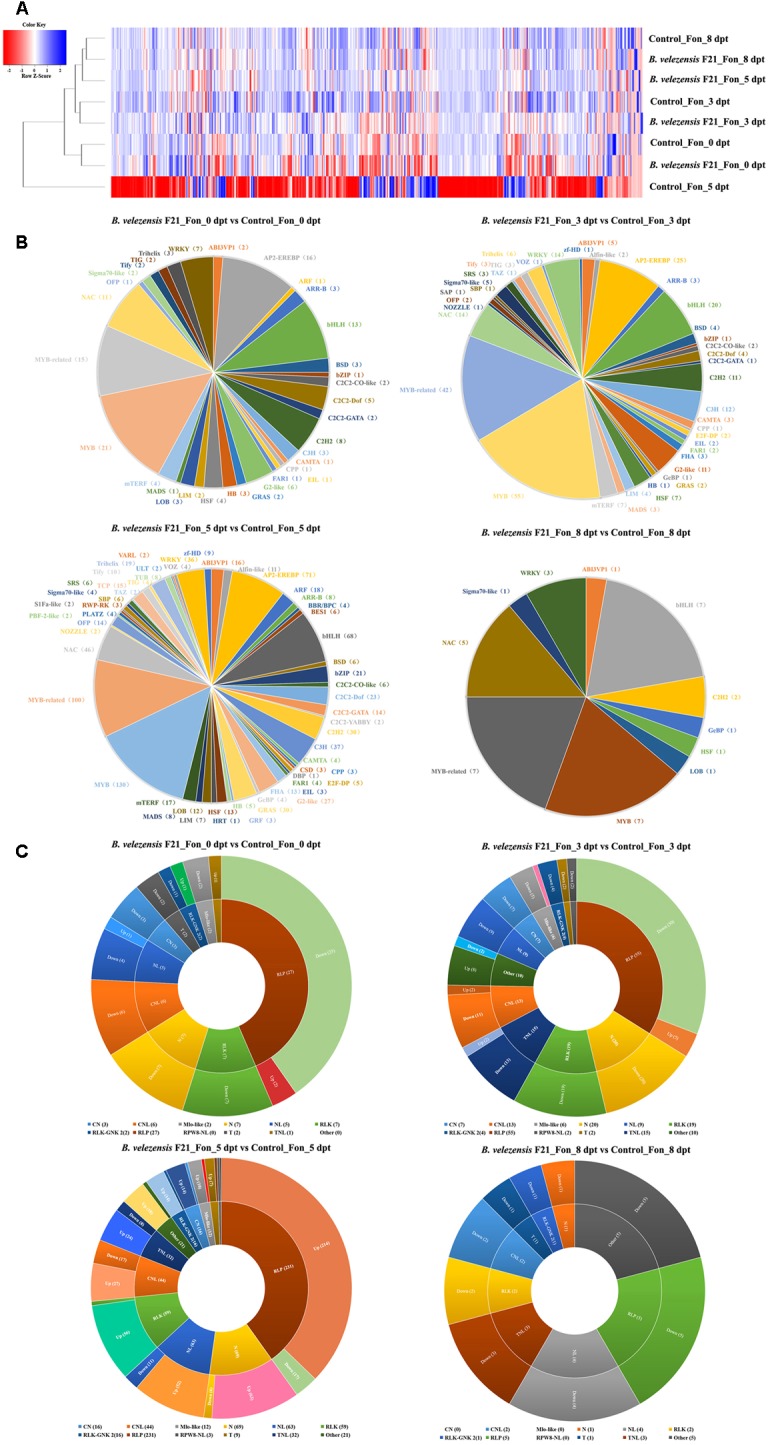
Transcription factors (TFs) and plant disease resistance genes prediction of DEGs. **(A)** Expression heat map of TFs coding DEGs in the comparisons of *B. velezensis* F21 pretreated or mock treated watermelon seedlings then both challenged with Fon at different timepoints. **(B)** DEGs classification on TFs family in the comparisons of watermelon seedlings in each treatment. **(C)** DEGs classification on plant disease resistance genes in the comparisons of watermelon seedlings in each treatment.

Transcription factors play important roles in plant growth and development, hormonal signaling, leaf senescence, and plant resistance to pathogens. To explore which TFs functioned on *B. velezensis* F21 triggered immunity to Fon, we analyzed the DEGs identified in each library. As shown in Supplementary Figure S6, *B. velezensis* F21 could affect the expression of TFs when treated with watermelon alone at different time points (Supplementary Figure S6A). Moreover, in the present study, a total of 36 kinds of TFs were identified when the watermelon was treated with *B. velezensis* F21 alone, including WRKY, MYB, bZIP, AP2, and NAC, which function in plant disease resistance. However, we also found that the types of TFs changed the most when watermelon was treated with *B. velezensis* F21 alone for 5 days (Supplementary Figure S6B). In addition, we also found that the expression of TFs was significantly influenced in the watermelons that were pretreated with *B. velezensis* F21 for 5 days and then challenged with Fon at different time points (Figure 6A). The numbers and types of TFs reached their maximum when challenged with Fon for 5 days, the number of types of TFs was 55. Similarly, it included some TFs related to plant disease resistance, such as MYB, WRKY, ERF, ARF, bZIP, AP2, and NAC (Figure 6B).

In addition, the numbers and kinds of PRGs were analyzed with the DEGs in each sample in our study as well. As shown in Supplementary Figure S7, eleven kinds of PRGs could be affected by *B. velezensis* F21, including CN, CNL, Mlo-like, N, NL, RLK, RLK-GNK, RLP, RPW0-NL, T and TNL, respectively. In addition, we also found that the PRGs could be influenced the most strongly, and almost 148 PRGs were identified when *B. velezensis* F21 alone was treated for 5 days (Supplementary Figure S7). Moreover, a total of 595 PRGs of eleven species were identified in the process of *B. velezensis* F21-triggered immunity to Fon, and the effect of PRGs reached a maximum when challenged with pathogens for 5 days (Figure 6C). The above results correspond to the expression intensity of defense-related genes and defense-related enzymes at the same time points as previously explored, and taken together, we could conclude that *B. velezensis* F21 could trigger plant immunity to Fon via enhancing the expression of TFs and PRGs.

### *B. velezensis* F21-Triggered Immunity to Fon Depended on the MAPK Signaling Pathway

**FIGURE 7:**
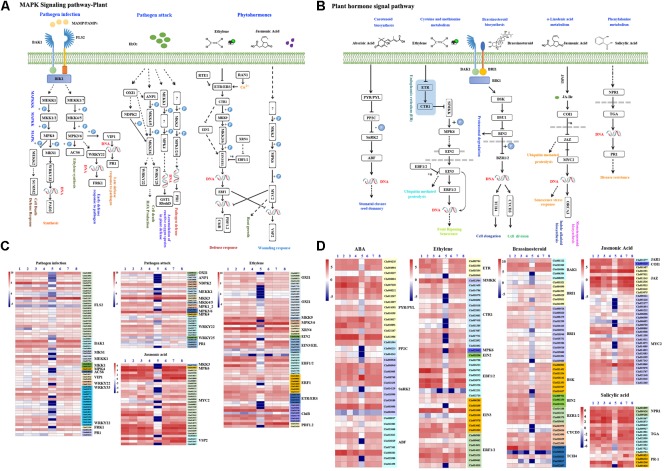
Analysis of DEGs related to MAPK signaling pathway and plant hormone signaling pathway. Analysis of DEGs related to MAPK signaling pathway and plant hormone signaling pathway in the comparisons of *B. velezensis* F21 pretreated or mock treated watermelon seedlings then both challenged with Fon. **(A)** The model of MAPK signaling pathway in plant. **(B)** The model of plant hormone signaling pathway in plant. **(C,D)** The heat map for expression of DEGs involved in MAPK signaling pathway and plant hormone signaling pathway, respectively, in the comparisons of *B. velezensis* F21 pretreated or mock treated watermelon seedlings then both challenged with Fon at different time points. The number 1, 3, 5, 7 means the samples got from the watermelon which was pretreated with sterile water then challenged with Fon at 0 dpt, 3 dpt, 5 dpt, and 8 dpt, respectively; the number 2, 4, 6, 8 means the samples got from the watermelon which was pretreated with *B. velezensis* F21 then challenged with Fon at 0 dpt, 3 dpt, 5 dpt, and 8 dpt, respectively.

Previous studies have demonstrated that the MAPK signaling pathway widely exists in eukaryotic organisms and participants in plant growth and responds to abiotic and biotic stress. As shown in Supplementary Figure S8A and Figure 7A, the MAPK signaling pathway response to plant pathogens could be divided into three parts, and a large number of genes were involved (Supplementary Figure S8A and Figure 7A). To investigate whether the MAPK signaling pathway was involved in *B. velezensis* F21-triggered immunity to Fon in watermelon, the above genes involved in the MAPK signaling pathway were analyzed with DEGs in each library. The results in Supplementary Figure S8B suggested that *B. velezensis* F21 could affect the expression of numerous of MAPK signaling pathway-related genes involved in plant responses to pathogen infection, pathogen attack and hormone. Moreover, we also found that the effect of *B. velezensis* F21 on the MAPK signaling pathway was the most significant when treated for 5 days (Supplementary Figure S8B). We also analyzed the expression levels of genes involved in the MAPK signaling pathway during the process of *B. velezensis* F21 triggering plant immunity to Fon. As shown in Figure 7C, numerous MAPK signaling pathway-related genes were influenced especially when the plants were pretreated with *B. velezensis* F21 and then challenged with Fon for 5 days (Figure 7C). The above results show that the MAPK signaling pathway plays a key role in *B. velezensis* F21 triggered plant immunity to Fon.

### Serials of Phytohormone Signaling Pathways Were Involved in *B. velezensis* F21 Triggered Immunity to Fon in Watermelon

Phytohormone signaling has been shown to have broad and crucial roles in plant development and response to biotic and abiotic stress. Therefore, in our study, we also studied the effect of *B. velezensis* F21 on changes in the phytohormone signaling pathway during the process of *B. velezensis* F21 triggering plant immunity to Fon. As shown in Supplementary Figure S9A and Figure 7B, many phytohormones participated in the plant immunity, such as ABA, ETH, BR, JA, and SA, and a large number of genes were involved in the synthesis and regulation of these phytohormones (Supplementary Figure S9A and Figure 7B). To validate which genes were involved in *B. velezensis* F21-triggered plant immunity to Fon, the expression of these genes was analyzed with the DEGs identified from transcriptome sequencing data. As shown in Supplementary Figure S9B, the numbers of genes involved in phytohormone synthesis and regulation were significantly affected by *B. velezensis* F21 (Supplementary Figure S9B). Likewise, the results in Supplementary Figure S8B demonstrated that the expression of these genes was influenced during *B. velezensis* F21-triggered plant immunity to Fon (Figure 7D). In addition, we also detected the accumulation of these phytohormones including ABA, ETH, BR, JA, and SA by ELISA in the process of *B. velezensis* F21-triggered plant immunity to Fon. It was found that the accumulation of these phytohormones in watermelon changed to varying degrees at different time points during the process of *B. velezensis* F21-triggered plant immunity to Fon (Supplementary Figure S10). The above results indicated that *B. velezensis* F21 could trigger plant immunity to Fon via activating the phytohormone signaling pathways.

To confirm the expression patterns identified by the transcriptome sequencing data of genes that were involved in the MAPK signaling pathway and phytohormones, the transcript levels of 15 genes during treatment with *B. velezensis* and 21 genes in the process of *B. velezensis* F21-triggered plant immunity to Fon were examined by qRT-PCR. The detailed information of the selected genes and primer pairs used in this study is shown in Supplementary Table S1. All the genes selected in this study were successfully amplified and the patterns of gene expression detected by qRT-PCR were consistent with those from the transcriptome sequencing data (Supplementary Figures S11, S12). Therefore, it turns out that the DEGs and gene expression profiles from transcriptome sequencing data were reliable.

## Discussion

Watermelon *Fusarium* wilt, caused by Fon, is a devastating disease affecting a large variety of economically important crops worldwide (Demers et al., 2015). The disease can result in 100% yield losses under favorable environmental conditions during watermelon production. At present, the control of the disease mainly depends on resistant varieties and chemical control, but the effect of the above control strategies is not ideal. PGPR were reported as an important functional group of beneficial bacteria used for plant growth promotion and the control of soilborne disease (Lemessa and Zeller, 2007). However, the exploitation of rhizobacteria as BCAs against this *Fusarium* wilt disease in watermelon has rarely been considered. In the present study, we found that *B. velezensis* F21 could serve as a potential BCA to control *Fusarium* wilt disease in watermelon. BCA not only promoted watermelon seedling growth (Supplementary Figure S2) but also significantly controlled *Fusarium* wilt disease in the greenhouse and filed (Figure 1C,D). However, the biocontrol of *Fusarium* wilt by *Bacillus* spp. has been reported in previously. For example, Rania et al. (2016) reported that *B. amyloliquefaciens* SP1 could significantly control *F. oxysporum*. However, *Fusarium* wilt disease in watermelon has rarely been considered. In the present study, we report for the first time that *B. velezensis* F21 could be used as a BCA for the control of *Fusarium* wilt disease. Moreover, we also found that *B. velezensis* F21showed remarkable production of many hydrolases (Supplementary Figure S1). Therefore, we hypothesized that this bacterium has strong and broad-spectrum bacteriostatic activity (Figure 1A).

In addition, as is well known, determining the mode of action is important and necessary for developing *Bacillus* sp. as a useful and successful BCA for the biocontrol of *Fusarium* wilt disease in watermelon (Jangir et al., 2018). In recent years, the focus was on the study of the antimicrobial activity of secondary metabolites. For example, Jangir et al. (2018) mentioned that one kind of *Bacillus* sp. B44, which was isolated from rhizospheric soil of tomato, could significantly protect against *F. oxysporum* f. sp. *lycopersici*, and study of the biocontrol mechanism indicated that the strain could produce numerous lytic enzymes and antifungal secondary metabolites (Jangir et al., 2018). Moreover, it was also reported that *B. pumilus* MSUA3 produced chitinolytic enzymes and utilized heat-stable antibiotic surfactin mediated antagonistic activity to control *F. oxysporum* (Agarwal et al., 2017). However, in our study, we demonstrated that *B. velezensis* F21 could not only directly suppress the growth of Fon by secreting some secondary metabolites with antimicrobial activity but also induce systemic resistance to Fon in watermelon. Many effective biocontrol PGPR have been reported to elicit ISR in recent years, including those that could elicit ISR to *Fusarium*. For instance, Duijff et al. (1998) investigated that *P. fluorescens* WCS417r could induce systemic resistance to suppress of *Fusarium* wilt of tomato. Otherwise, some non-pathogen fungi, such as *Phytophthora cryptogea*, were also reported to have the ability to induce systemic resistance to Fon in the host (Attitalla et al., 2010). In our study, as shown in Figure 2, 3, we found that the transcripts of two tested genes, *Cla01114 and Cla005426*, were both generally stronger in the roots of watermelon pretreated with *B. velezensis* F21 and then challenged with Fon from 3 dpt to 9 dpt and reached their maximums at 3 dpt compared with the control treatment. Moreover, the activities of defense-related enzymes (CAT, POD, and SOD) were also determined and the results indicated that compared with the control treatment, *B. velezensis* F21 could increase the activities and advance those defense-related enzymes to maximum activity in advance in response to Fon invasion. Taken together, we conclude that *B. velezensis* F21 could prime the watermelon to enhance resistance to Fon by simultaneously activating the SA-and JA-mediated defense signaling pathways. This is also the first report that *B. velezensis* can control *Fusarium* wilt disease by inducing systemic resistance to Fon in watermelon by simultaneously activating the SA-and JA-mediated defense signaling pathways.

Recent studies show that the application of transcriptomics can provide precise information on gene networks for identifying the functions of genes and unknown genes on regulation (Tohge et al., 2005). In this study, we identified thousands of genes that function on *B. velezensis* F21 ISR to Fon in watermelon. As shown in Supplementary Table S4 and Figure 3A,B, the expression of a large number of genes in watermelon could be changed or modified by *B. velezensis* F21 treated alone or pretreated before the inoculation of pathogens (Supplementary Table S4 and Figure 3A,B), indicating that *B. velezensis* F21 could induce systemic resistance to Fon via altering transcriptional levels of genes in watermelon. In addition, from the results, we hypothesize that there must be many genes involved in regulation in this process. TFs have been demonstrated to play essential roles in plants by controlling the expression of genes involved in various cellular processes (Han et al., 2014; Chen F. et al., 2017). It consists of many families, such as WRKY, AP2-ERF, MYB, and NAC. WRKY factors have been verified to fulfill essential regulatory functions to modulate pathogen-triggered cellular responses in a number of plant species, and most WRKY factors participate in the SA signaling pathway (Tsuda and Somssich, 2015; Chen F. et al., 2017). However, some ERF factors have been demonstrated, which participate in the JA signaling pathway are involved in the interaction between plants and insects of some fungal pathogens (Mizoi et al., 2012). In addition, NAC proteins are another kind of plant-specific TF that constitute a large family. At present, many functional studies have shown that NAC TFs could regulate immune responses in plants, and over 100 NAC genes have been identified in Arabidopsis (Puranik et al., 2012; Lee et al., 2017). In our study, by comparing the RNA sequencing data, we also identified numerous TFs involved in the process of *B. velezensis* F21inducing systemic resistance to Fon in watermelon, including WRKY, MYB, bZIP, AP2, and NAC (Supplementary Figure S6 and Figure 6A,B). Moreover, we found that two families were both identified in our sequencing data, WRKY and AP2-ERF. The results indicated that the two signaling pathways, both SA and JA, take part in the process of *B. velezensis* F21 inducing systemic resistance to Fon in watermelon. The above results coincide with the conclusion of a previous expression level of gene determination by qRT-PCR.

Plant MAPKs are involved in plant growth, development, responses to endogenous and environmental cues. Serials of studies have demonstrated that MAPKs could be activated by external sensors to cellular responses (Yu and Tang, 2004). Otherwise, some studies verified that MAPKs were involved in the interaction between plant and pathogens, and they played key roles in plant response to pathogen invasion. For instance, many effectors secreted via pathogens have been found to inhibit MAP kinase cascade (Bi and Zhou, 2017). In our study, we also analyzed the transcriptional levels of genes related to MAP kinase cascade, and as the results shown in Supplementary Figure S8, many genes were affected by *B. velezensis* F21, this indicated that the perception of *B. velezensis* F21 in watermelon also depends on MAPK pathway. From the result shown in Figure 7C, we could demonstrate that the pretreatment of *B. velezensis* F21 could speed up the recognition of pathogens in plants, make corresponding resistance response quickly, and enhance the immunity of plants. The above hypothesis is only based on the results of current bioinformatics analysis. In-depth analysis and verification work needs to be completed in future research.

Phytohormones are some substances biosynthesized in very low concentrations but able to regulate serials of cellular processes in plants. They play key roles and coordinate various signal transduction pathways during abiotic and abiotic-stress response (Voß et al., 2014). For instance, some phytohormones, such as ABA, JA, ETH, SA, etc., have been identified as stress hormones. They played critical roles in plant growth regulation, development, stomatal closure and mediating abiotic and biotic stress responses (Li et al., 2010). In this study, we also evaluated the effect of *velezensis* F21 on phytohormones synthesis and regulation. Therefore, we analyzed the expression level of some genes involved in some phytohormones and detected the accumulation of these phytohormones in watermelon at different treatment by ELISA, such as ABA, JA, ETH, SA, and so on. As the results shown in Figure 7D and Supplementary Figures S9, S10, number of genes involve in phytohormones synthesis and regulation was significantly affected by *B. velezensis* F21. Moreover, we also found that the accumulation of these phytohormones in watermelon changed in varying degrees at different time points during the process of *B. velezensis* F21 triggered plant immunity to Fon. These results suggested that phytohormones in watermelon played critical roles in *B. velezensis* F21 triggered plant immunity to Fon. However, it still requires us to invest more time in future work to figure out how the BCAs regulate phytohormone synthesis and which kind of phytohormones play the main roles in *B. velezensis* F21 triggered plant immunity to Fon.

## Conclusion

In this study, we identified an efficiently PGPR strain *B. velezensis* F21, which could be used for control of Fon in watermelon. In-depth mechanism study indicated that *B. velezensis* F21 could suppress the growth and spore germination of Fon *in vitro*. Moreover, *B. velezensis* F21 could also enhance plant basal immunity to Fon by increasing the expression of plant defense related genes and activities of some defense enzymes, such as CAT, POD, and SOD. In addition, a comparative transcriptome analysis was employed to figure out more detail mechanisms and the results revealed almost thousands of DEGs in the process of *B. velezensis* F21 triggered ISR to Fon, among these identified DEGs, a large number of genes belong to some TFs, plant resistant genes, some genes involved in MAP kinase cascade and phytohormone biosynthesis and regulation. Taken together, this study substantially expands transcriptome data resources and suggests a molecular framework for *B. velezensis* F21 inducing systemic resistance to Fon in watermelon. It also provides a new idea for the mechanism study to understand how BCAs biocontrol *Fusarium* wilt disease in watermelon. Besides, it also provides an effective strategy for the control of *Fusarium* wilt in watermelon.

## Author Contributions

C-HJ, X-FY, and J-HG designed the experiments. D-DM, Z-JL, and B-YY performed the experiments with assistance from YZ, C-HJ, Y-JQ, and J-HG analyzed and discussed the results. C-HJ wrote the manuscript.

## Conflict of Interest Statement

The authors declare that the research was conducted in the absence of any commercial or financial relationships that could be construed as a potential conflict of interest.

## Footnotes

1 http://trinityrnaseq.github.io/

2 http://plntfdb.bio.uni-potsdam.de

3 http://prgdb.crg.eu/wiki/Main_Page

4 https://elisa.biomart.cn

## Abbreviations

ABA: abscisic acid
BIO: bio-organic fertilizer
BR: brassinosteroid
CAT: catalase
DEGs: differentially expressed genes
ELISA: enzyme-linked immunosorbent assay
ETH: ethylene
Fon: *Fusarium oxysporum* f. sp. *niveum*
GO: Gene Ontology
ISR: induced systemic resistance
JA: jasmonic acid
KEGG: Kyoto Encyclopedia of Genes and Genomes
LB medium: Luria-Bertani medium
MAPK: mitogen-activated protein kinase
PDA medium: potato dextrosa agar medium
PGPR: plant growth promotion rhizobacteria
POD: peroxidase
SA: salicylic acid
SOD: superoxide dismutase
TFs: transcription factors
