# Supplementary material for: Comparative Transcriptome Analysis Reveals the Biocontrol Mechanism of Bacillus velezensis F21 Against Fusarium Wilt on Watermelon
Source: Front Microbiol. 2019 Apr 3;10:652. doi: 10.3389/fmicb.2019.00652 (PMC6456681; doi:10.3389/fmicb.2019.00652)
Supplement: Supplementary file 5 [file Data_Sheet_1.docx]

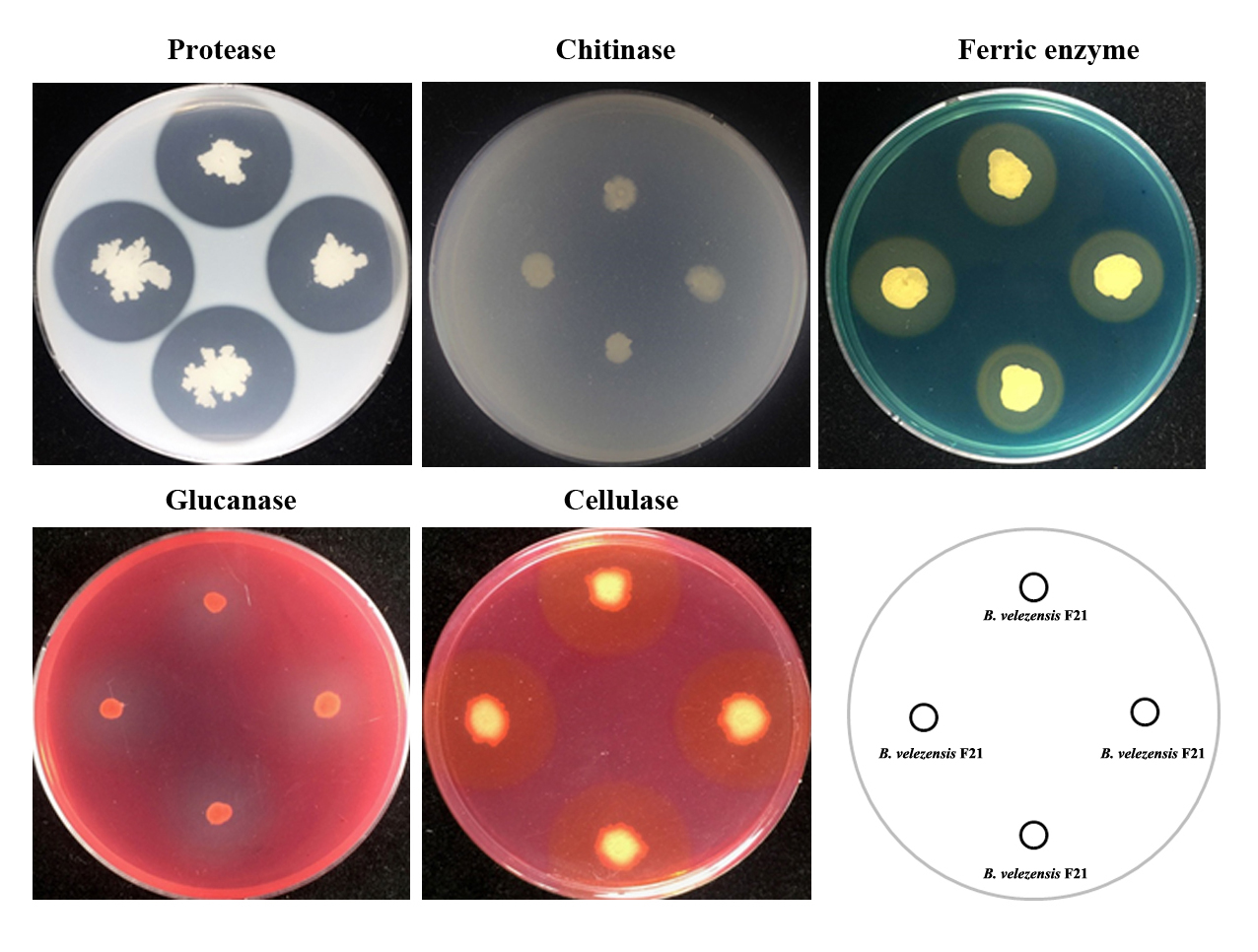


**Figure S1. Determination of hydrolase activity of beneficial rhizobacterium *B. velezensis* F21.**

Note: Bacteria hydrolase such as protease; chitinase; ferric enzyme; glucanase and cellulase, were evaluated on the special medium.


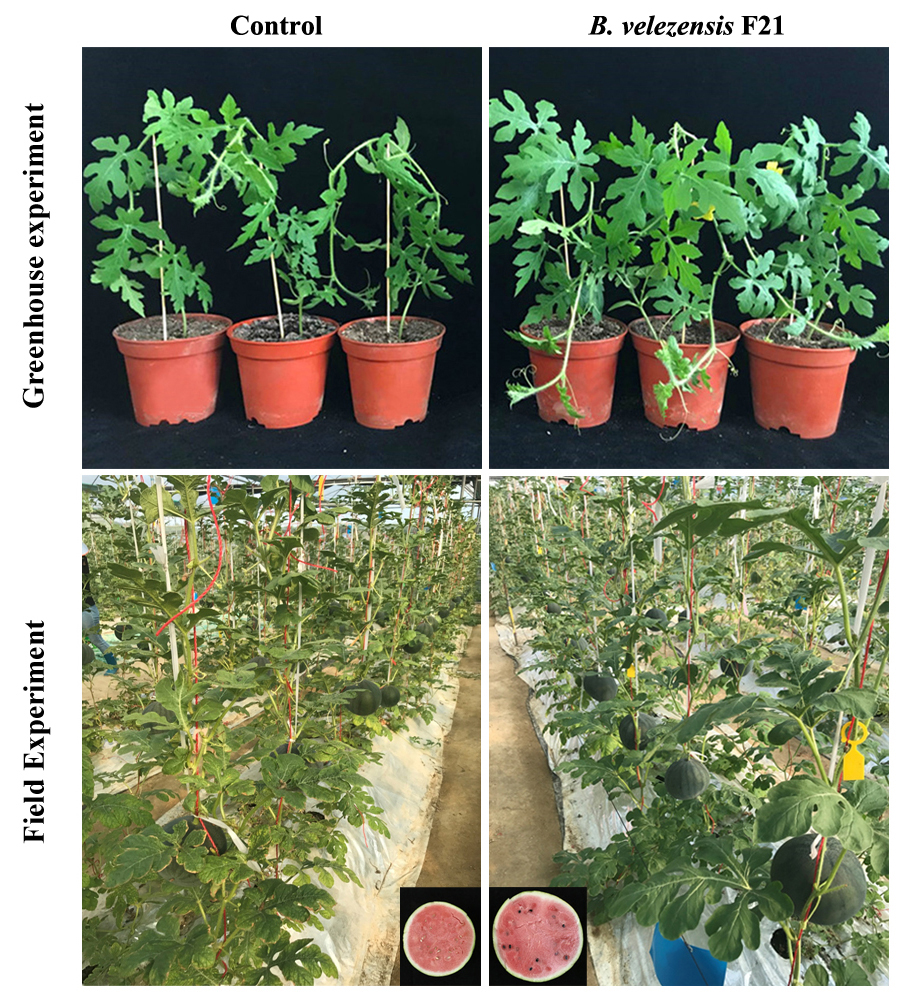


**Figure S2. The growth-promoting effect of *B. velezensis* F21 on watermelon.**

Note: The Effects of beneficial rhizobacterium *B. velezensis* F21 on the growth promotion to watermelon in greenhouse experiments and field experiments respectively.


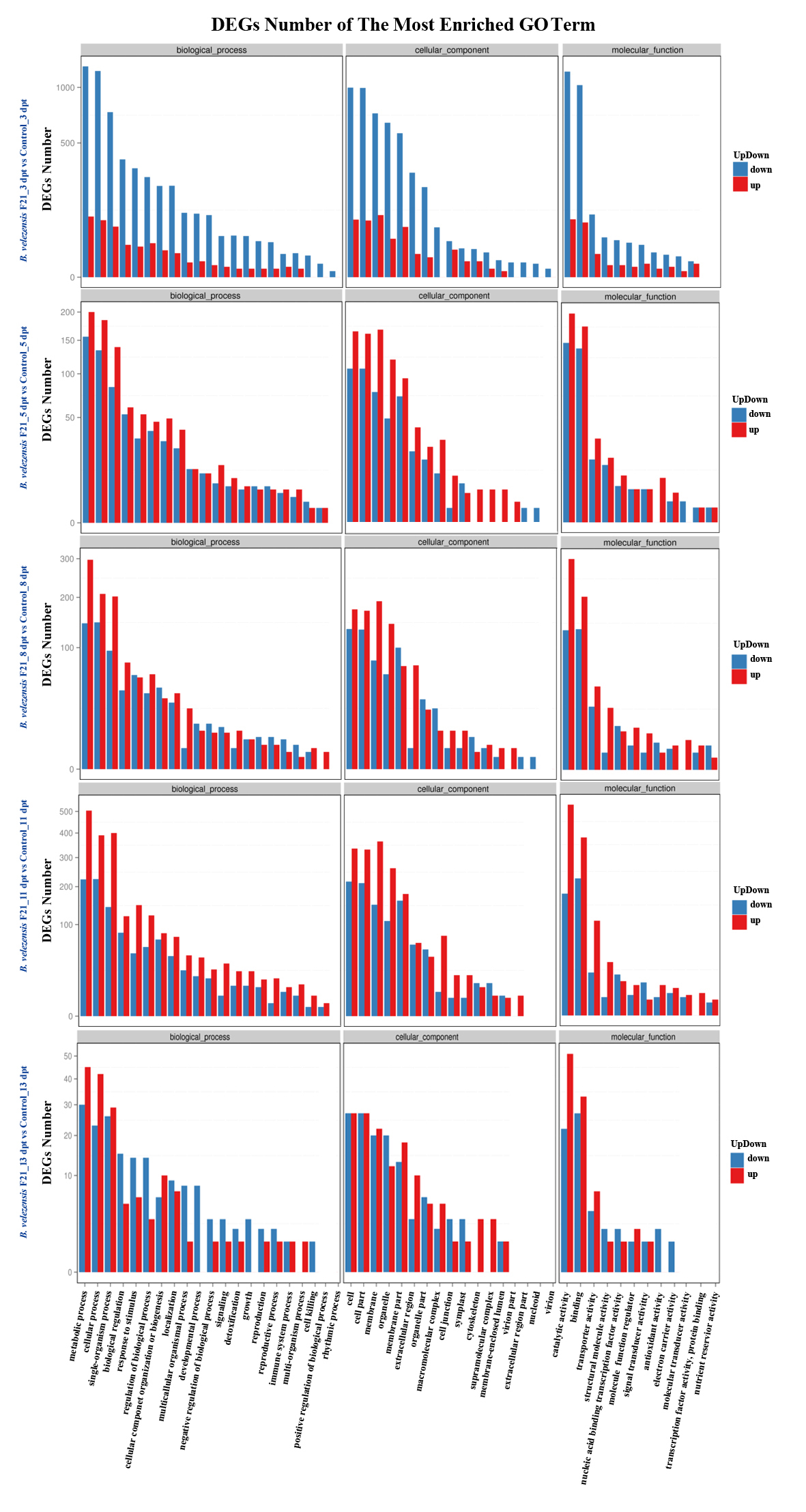


**Figure S3. GO classification of up-regulated and down-regulated genes identified from the watermelon seedlings treated with *B. velezensis* F21 alone.**

Note: GO classification of DEGs in the comparisons of *B. velezensis* F21 treated alone and mock treated watermelon seedlings at different time points. X axis represents GO term. Y axis represents the amount of up/down-regulated genes.


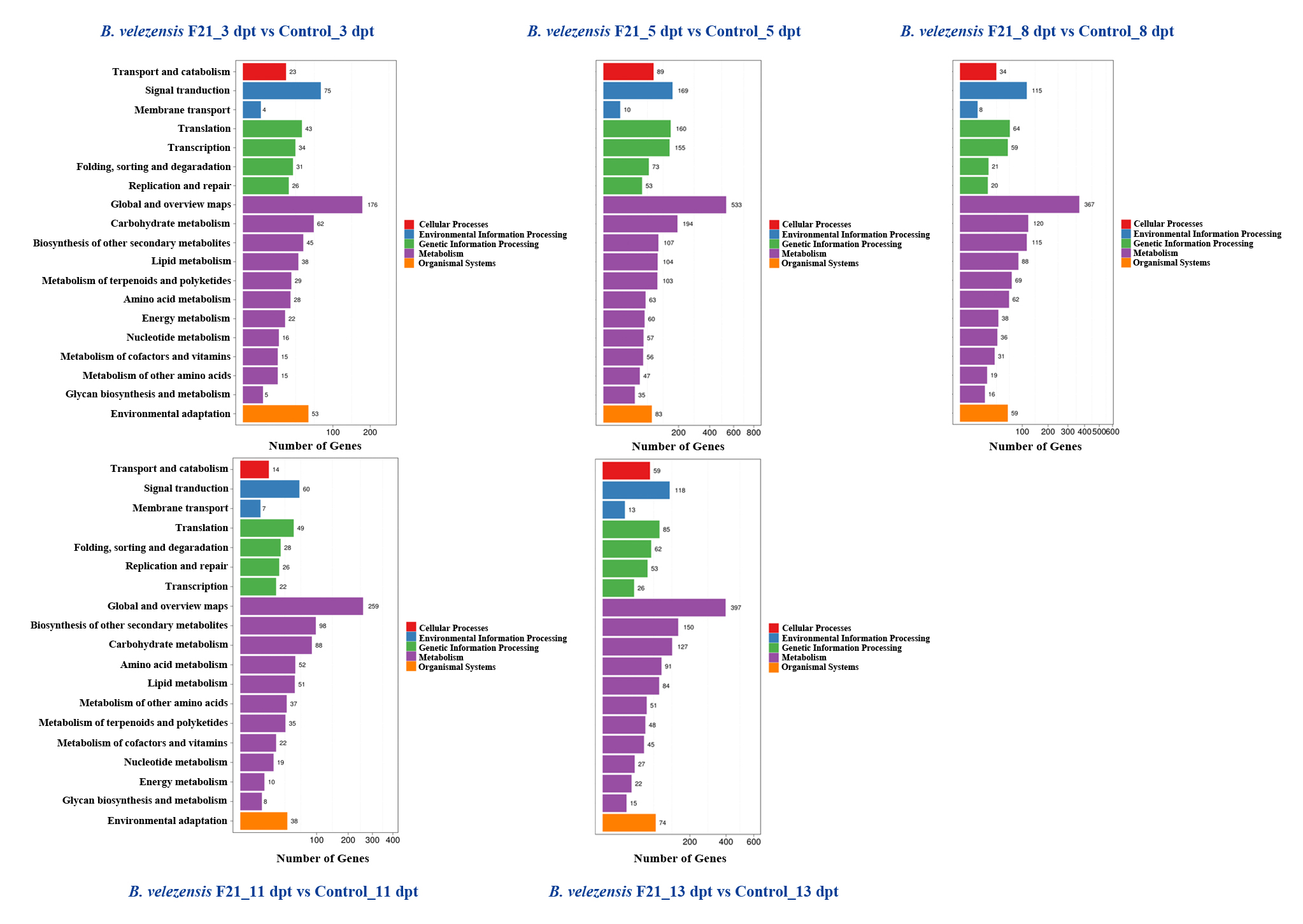


**Figure S4. Kyoto Encyclopedia of Genes and Genomes (KEGG) Pathway classification of DEGs identified from the watermelon seedlings treated with *B. velezensis* F21 alone.**

Note: Pathway classification of DEGs in the comparisons of *B. velezensis* F21 treated alone and mock treated watermelon seedlings at different time points, X axis represents number of DEG. Y axis represents functional classification of KEGG. There are seven branches for KEGG pathways: Cellular Processes, Environmental Information Processing, Genetic Information Processing, Human Disease (For animals only), Metabolism, Organismal Systems and Drug Development.


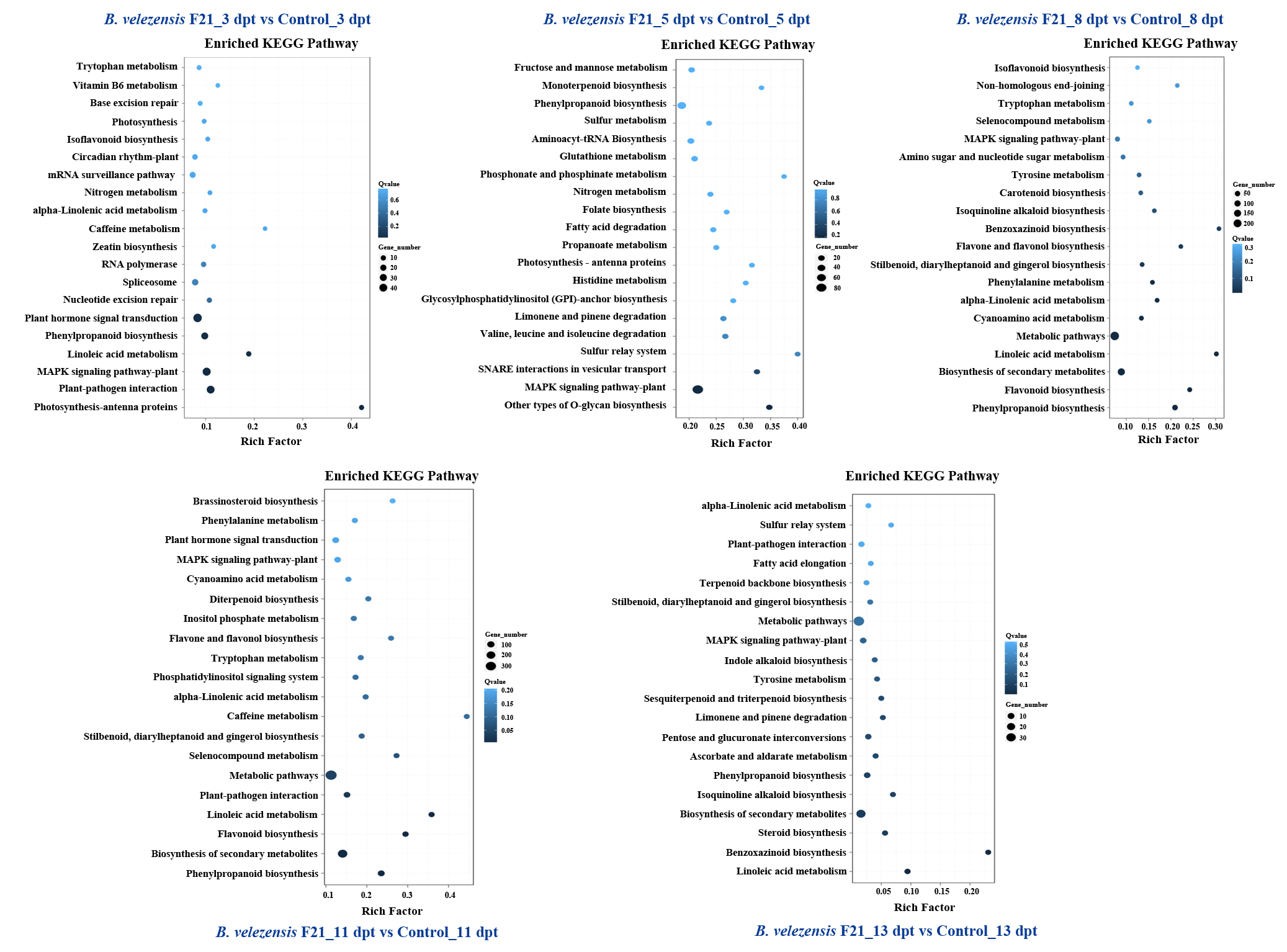


**Figure S5. Kyoto Encyclopedia of Genes and Genomes (KEGG) Pathway functional enrichment of DEGs identified from the watermelon seedlings treated with *B. velezensis* F21 alone.**

Note: Pathway functional enrichment of DEGs in the comparisons of *B. velezensis* F21 treated alone and mock treated watermelon seedlings at different time points, X axis represents enrichment factor. Y axis represents pathway name. The color indicates the q-value (high: white, low: blue), the lower q-value indicates the more significant enrichment. Point size indicates DEG number (The bigger dots refer to larger amount). Rich Factor refers to the value of enrichment factor, which is the quotient of foreground value (the number of DEGs) and background value (total Gene amount). The larger the value, the more significant enrichment.


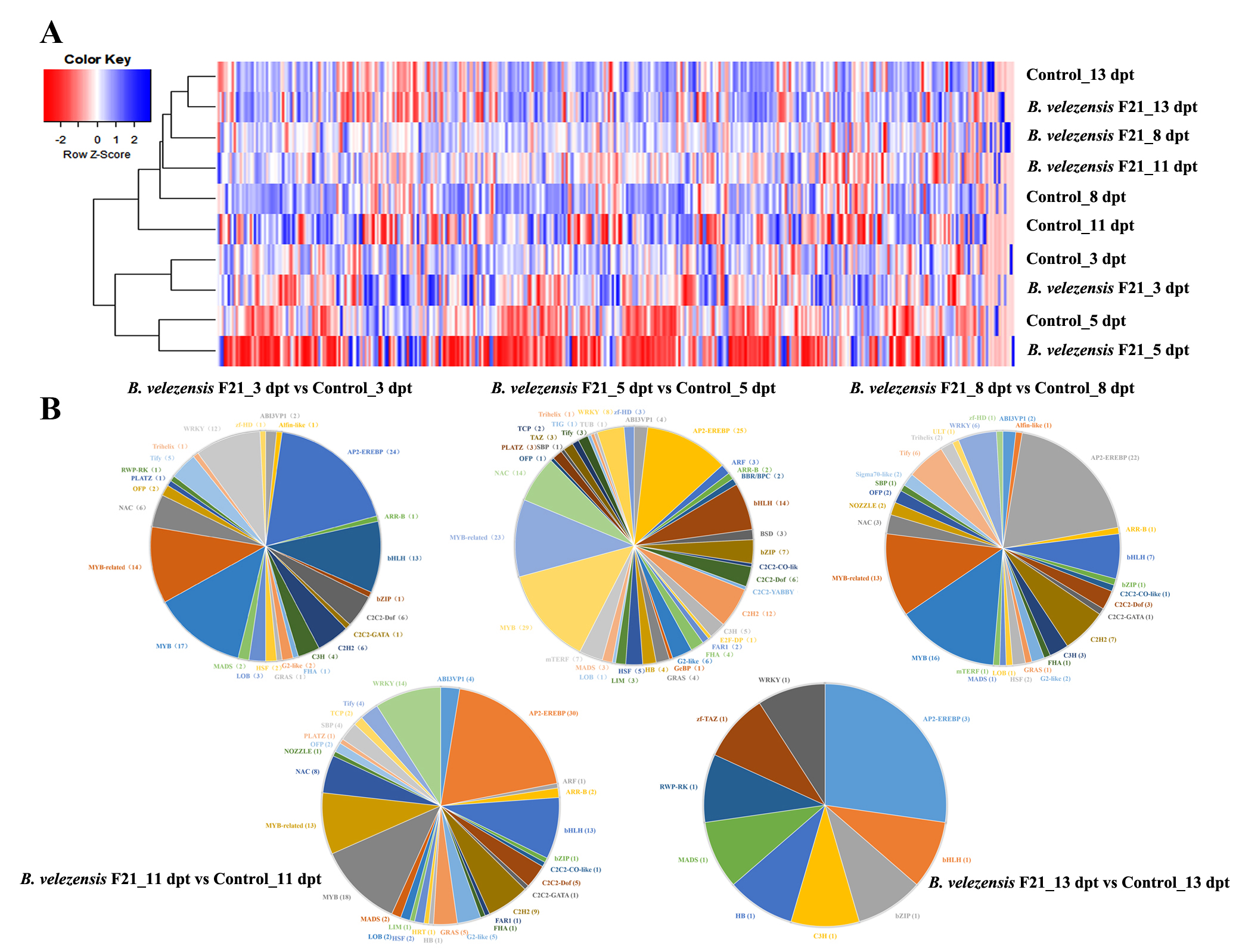


**Figure S6. Transcription Factors (TFs) Prediction of DEGs identified from the watermelon seedlings treated with *B. velezensis* F21 alone.**

Note: (A) Expression heat map of TFs coding DEGs in the comparisons of *B. velezensis* F21 treated alone and mock treated watermelon seedlings; (B) DEGs classification on TFs family in the comparisons of watermelon seedlings in each treatment.


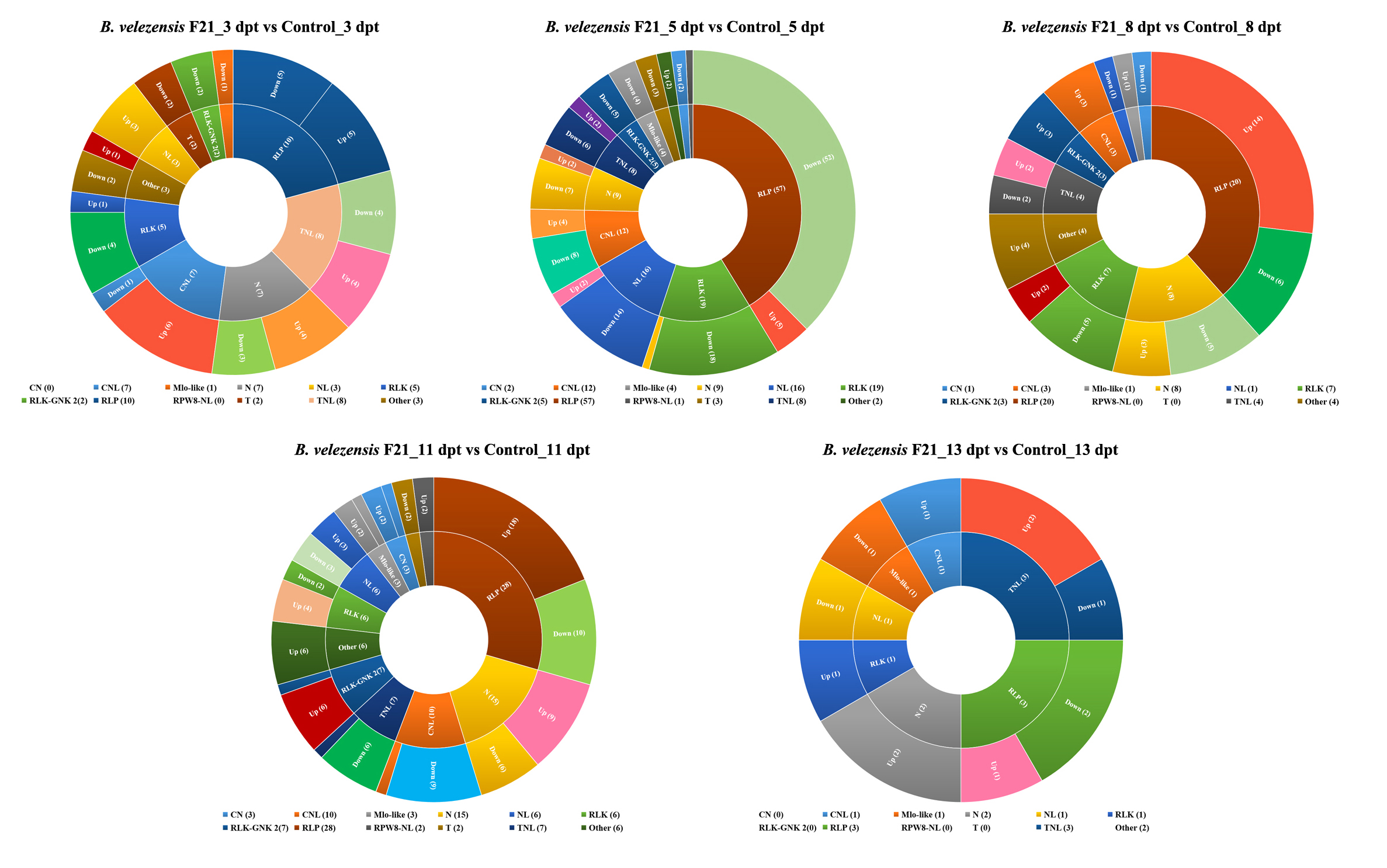


**Figure S7. Plant Disease Resistance Genes Prediction of DEGs identified from the watermelon seedlings treated with *B. velezensis* F21 alone.**

Note: DEGs classification on Plant Disease Resistance Genes in the comparisons of watermelon seedlings treated alone and mock treated watermelon seedlings.


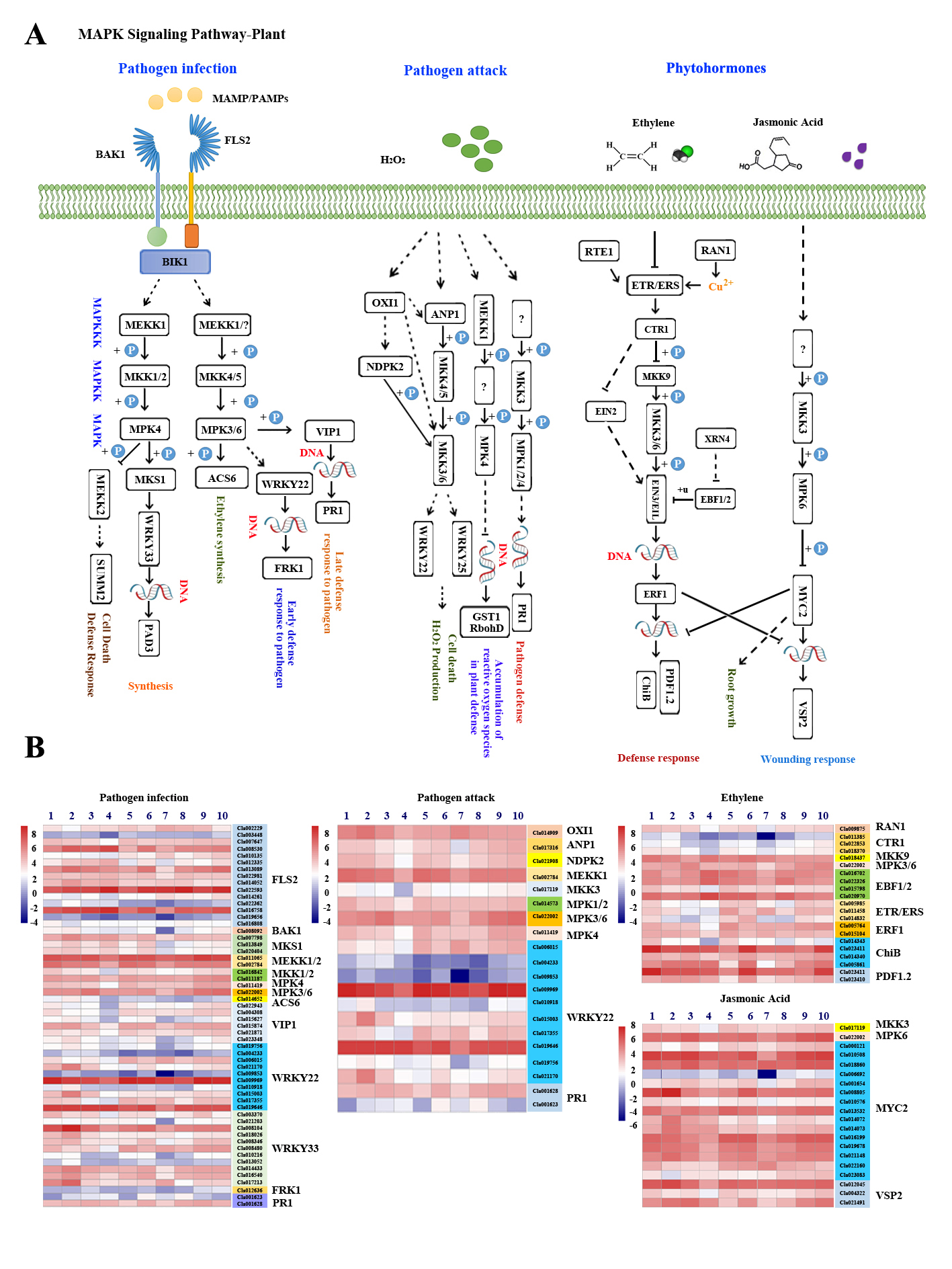


**Figure S8. Analysis of DEGs related to MAPK signaling pathway in the comparisons of watermelon seedlings treated with *B. velezensis* F21 alone**

Note: (A) The model of MAPK signaling pathway in plant; (B) The heat map for expression of DEGs involved in MAPK signaling pathway in the comparisons of *B. velezensis* F21 treated alone at different time points. The number 1, 3, 5, 7, 9 means the samples got from the watermelon which was treated with sterile water alone at 3 dpt, 5 dpt, 8 dpt, 11 dpt and 13 dpt respectively; the number 2, 4, 6, 8, 10 means the samples got from the watermelon which was treated with *B. velezensis* F21 alone at 3 dpt, 5 dpt, 8 dpt, 11 dpt and 13 dpt respectively.

**Figure S9. Analysis of DEGs related to plant hormone signaling pathway in the comparisons of watermelon seedlings treated with *B. velezensis* F21 alone**


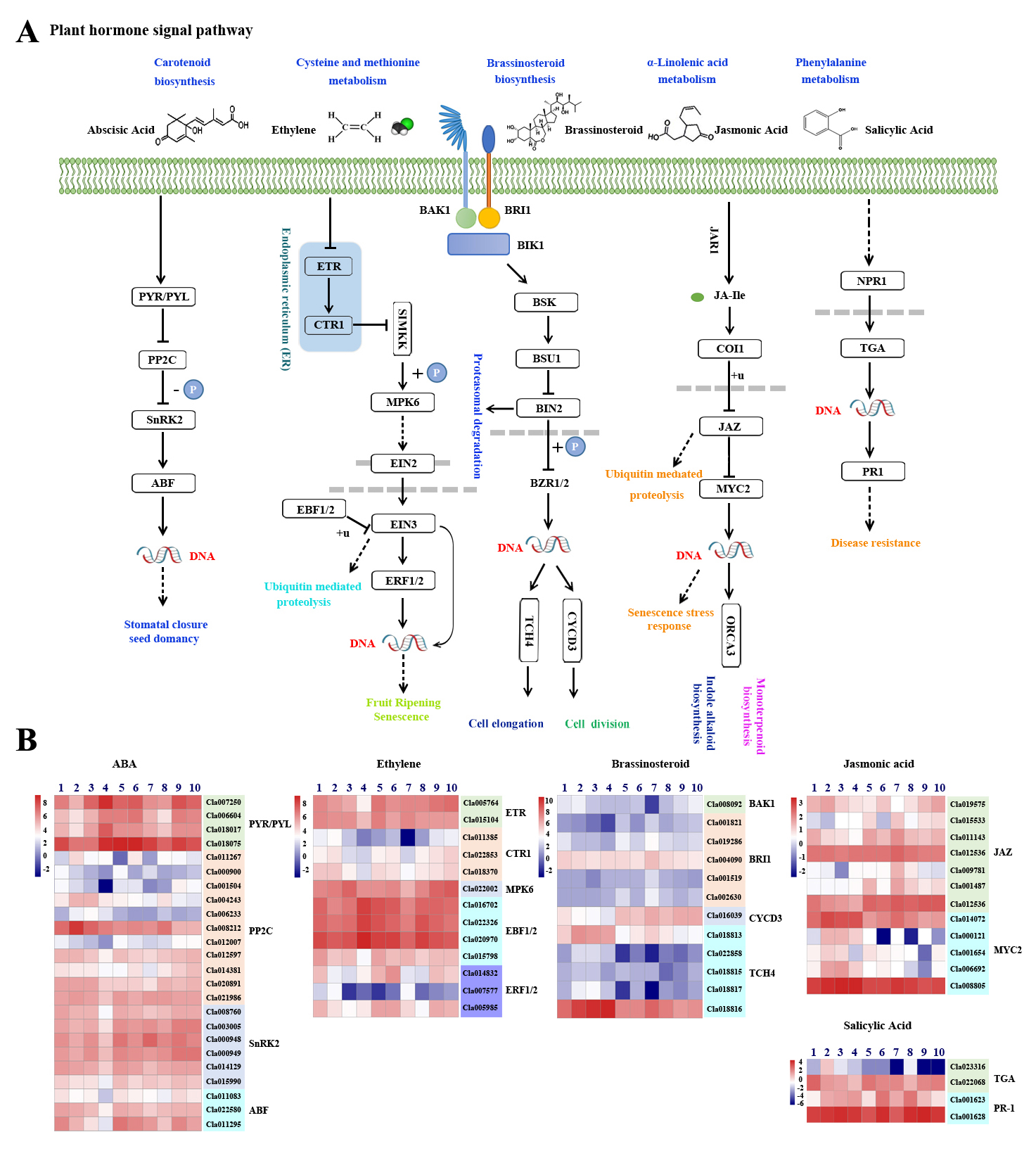


Note: (A) The model of plant hormone signaling pathway in plant; (B) The heat map for expression of DEGs involved in plant hormone signaling pathway in the comparisons of *B. velezensis* F21 treated alone at different time points. The number 1, 3, 5, 7, 9 means the samples got from the watermelon which was treated with sterile water alone at 3 dpt, 5 dpt, 8 dpt, 11 dpt and 13 dpt respectively; the number 2, 4, 6, 8, 10 means the samples got from the watermelon which was treated with *B. velezensis* F21 alone at 3 dpt, 5 dpt, 8 dpt, 11 dpt and 13 dpt respectively.


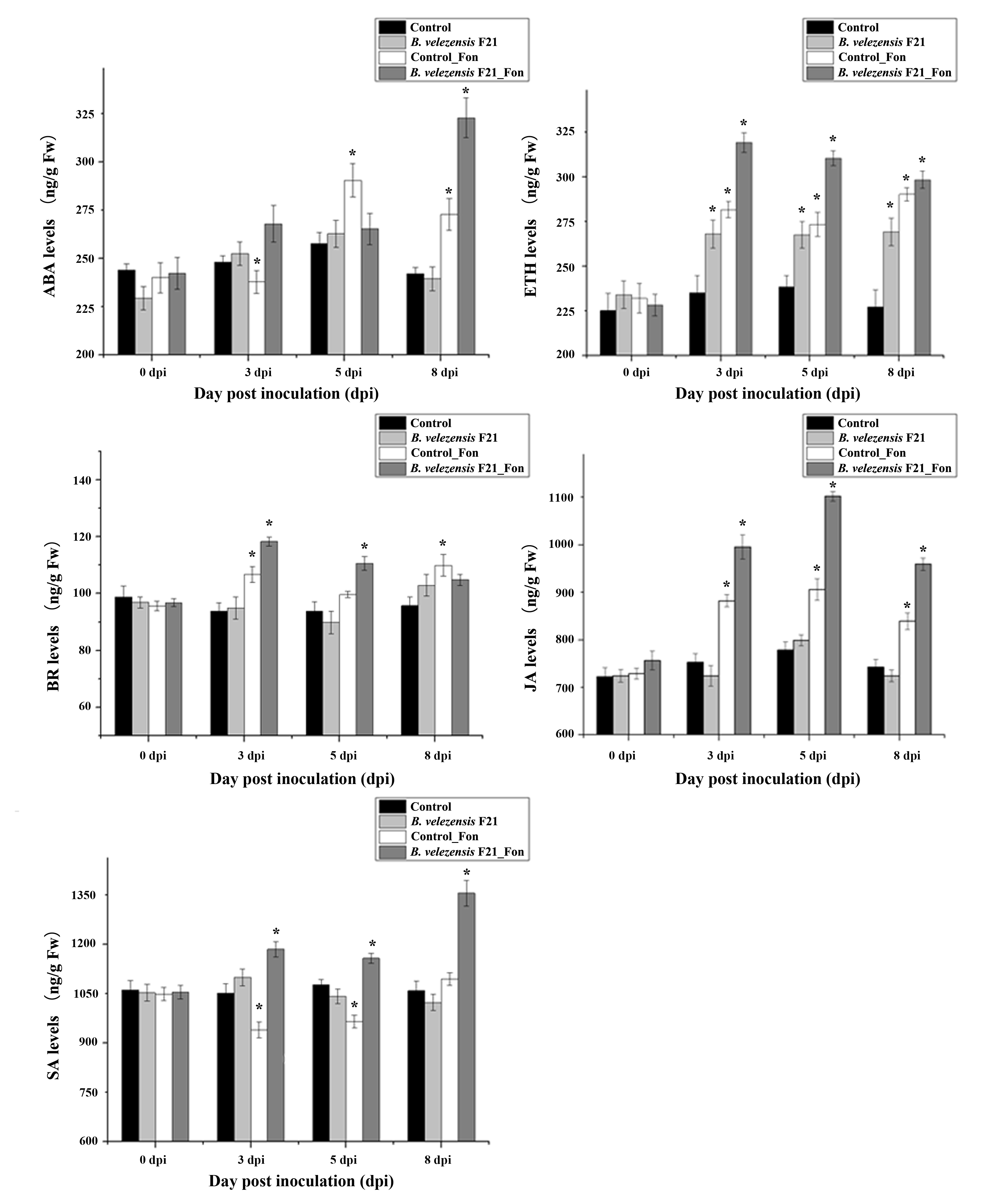


**Figure S10. Time course of phytohormones accumulation in root of watermelon pretreated with *B. velezensis* F21 then challenged with Fon.**

Note: Endogenous levels of five phytohomones (ABA, BR, ETH, SA and JA) in root of watermelon at different time points were detected by ELISA Kit. All Data were presented as means of three replicates ± SD, and error bars represent SD for three replicates. Means with asterisk have significant differences (*: P < 0.05; LSD test). All experiments were performed three times, and similar results were obtained.


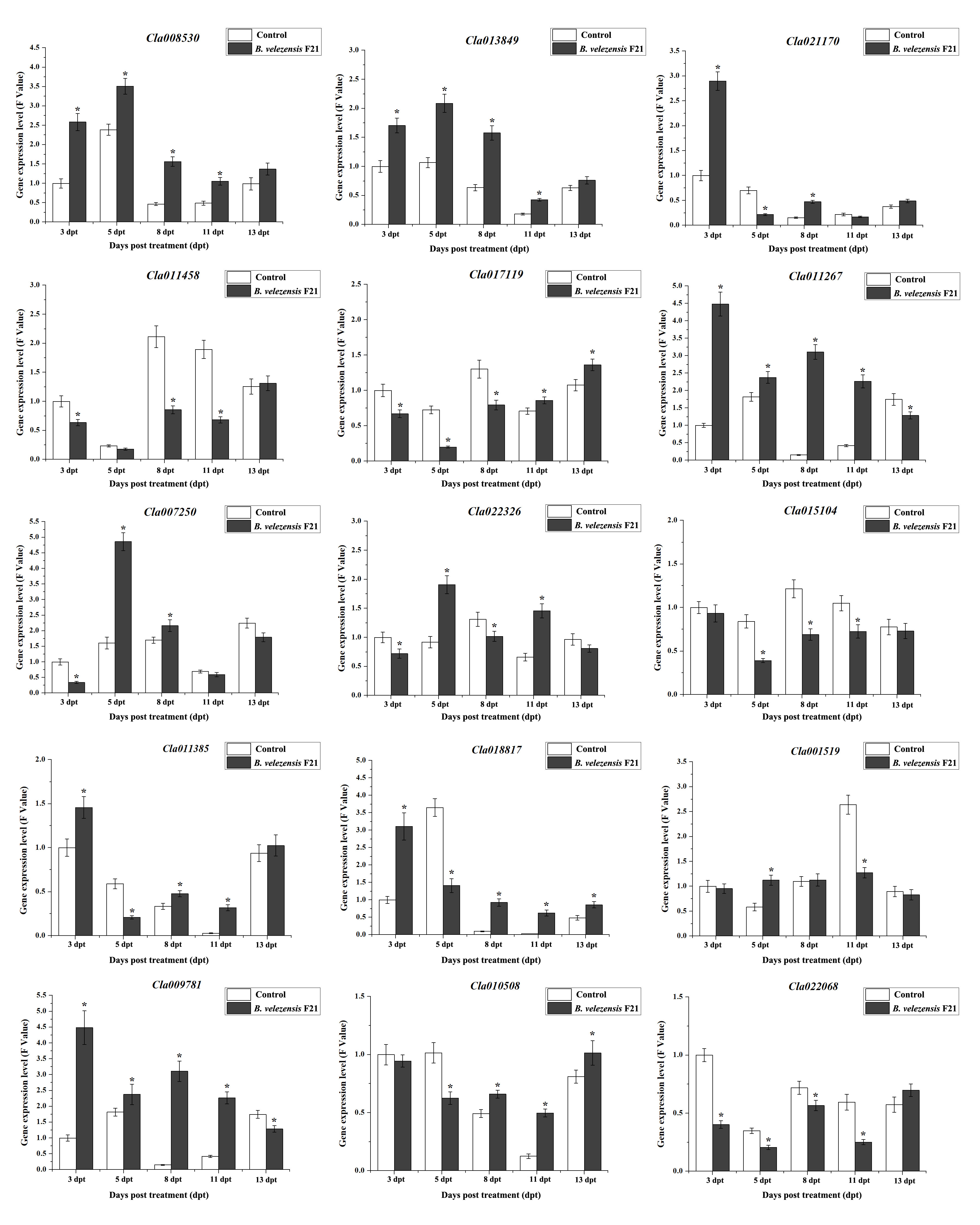


**Figure S11. Validation of the expression proﬁle of transcriptome sequencing data from watermelon seedlings treated with *B. velezensis* F21 alone by Q-RT-PCR.**

Note: Fifteen genes which were related on plant resistance and phytohormones synthesis were selected and validated by Q-RT-PCR to conﬁrm their expression proﬁles determined by RNA-Seq in the watermelon root treated by *B. velezensis* F21 alone.

**Figure S12. Validation of the expression proﬁle of transcriptome sequencing data from watermelon pretreated with *B. velezensis* F21 then challenged with Fon by Q-RT-PCR.**


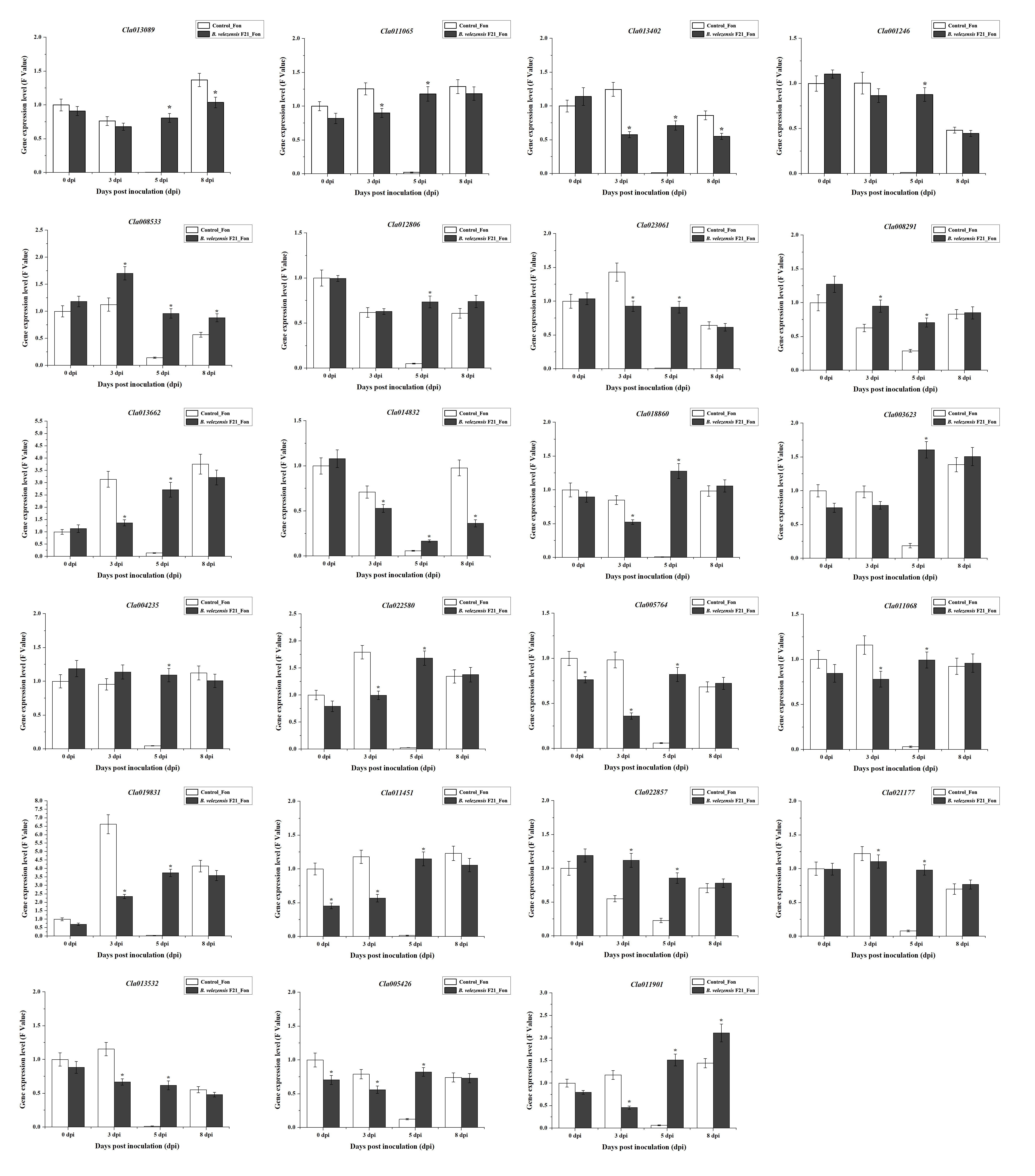


Note: Twenty-three genes which were related on plant resistance and phytohormones synthesis were selected and validated by Q-RT-PCR to conﬁrm their expression proﬁles determined by RNA-Seq in the root of watermelon pretreated with *B. velezensis* F21 and challenged with Fon.
